# Supplementary figures and images for: Retrospective analysis assessing the spatial and temporal distribution of paediatric acute respiratory tract infections in Ho Chi Minh City, Vietnam
Source: BMJ Open. 2018 Jan 21;8(1):e016349. doi: 10.1136/bmjopen-2017-016349 (PMC5780701; doi:10.1136/bmjopen-2017-016349)

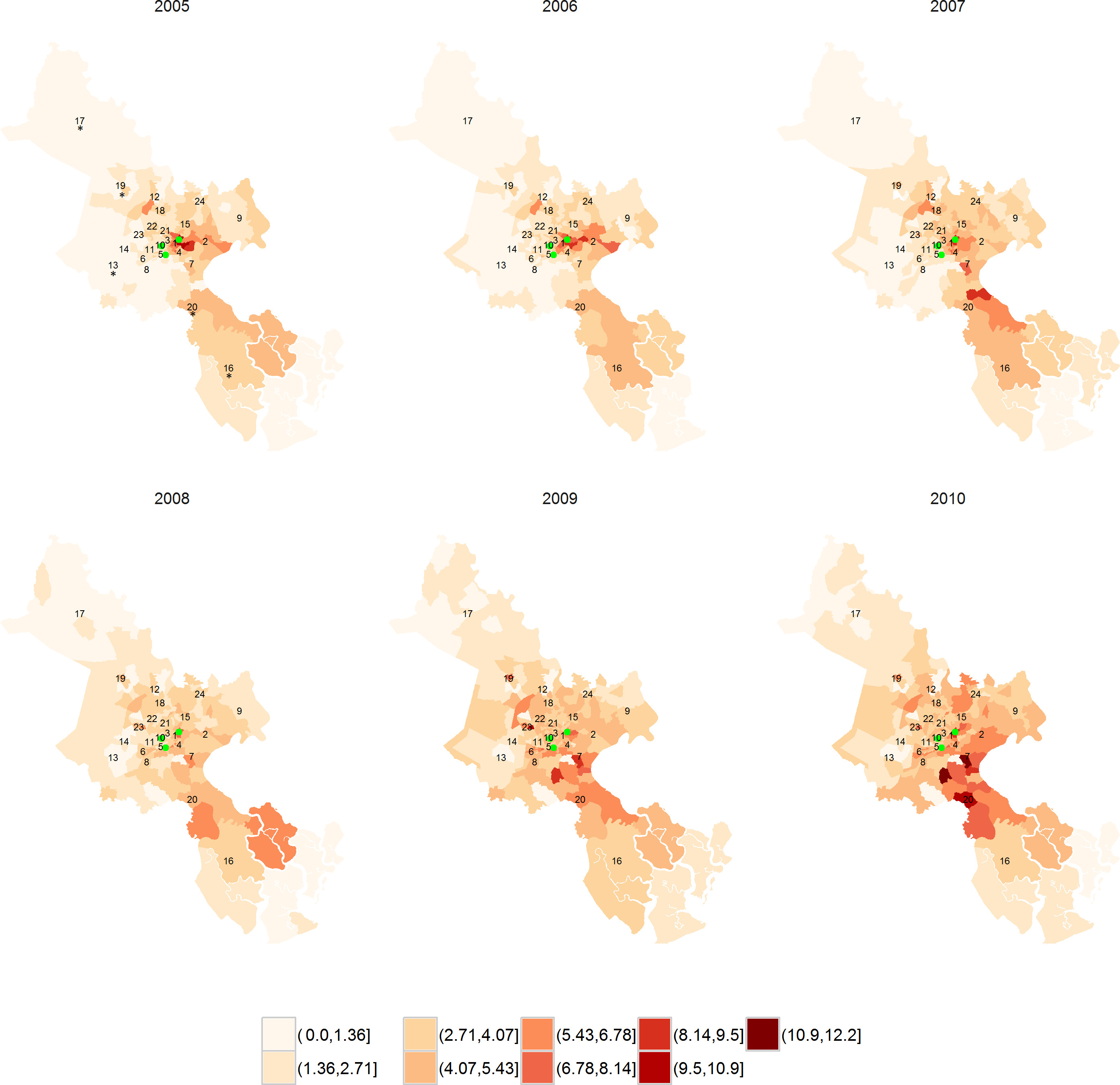

Supplement: Supplementary file 1 [file bmjopen-2017-016349supp001.jpg]

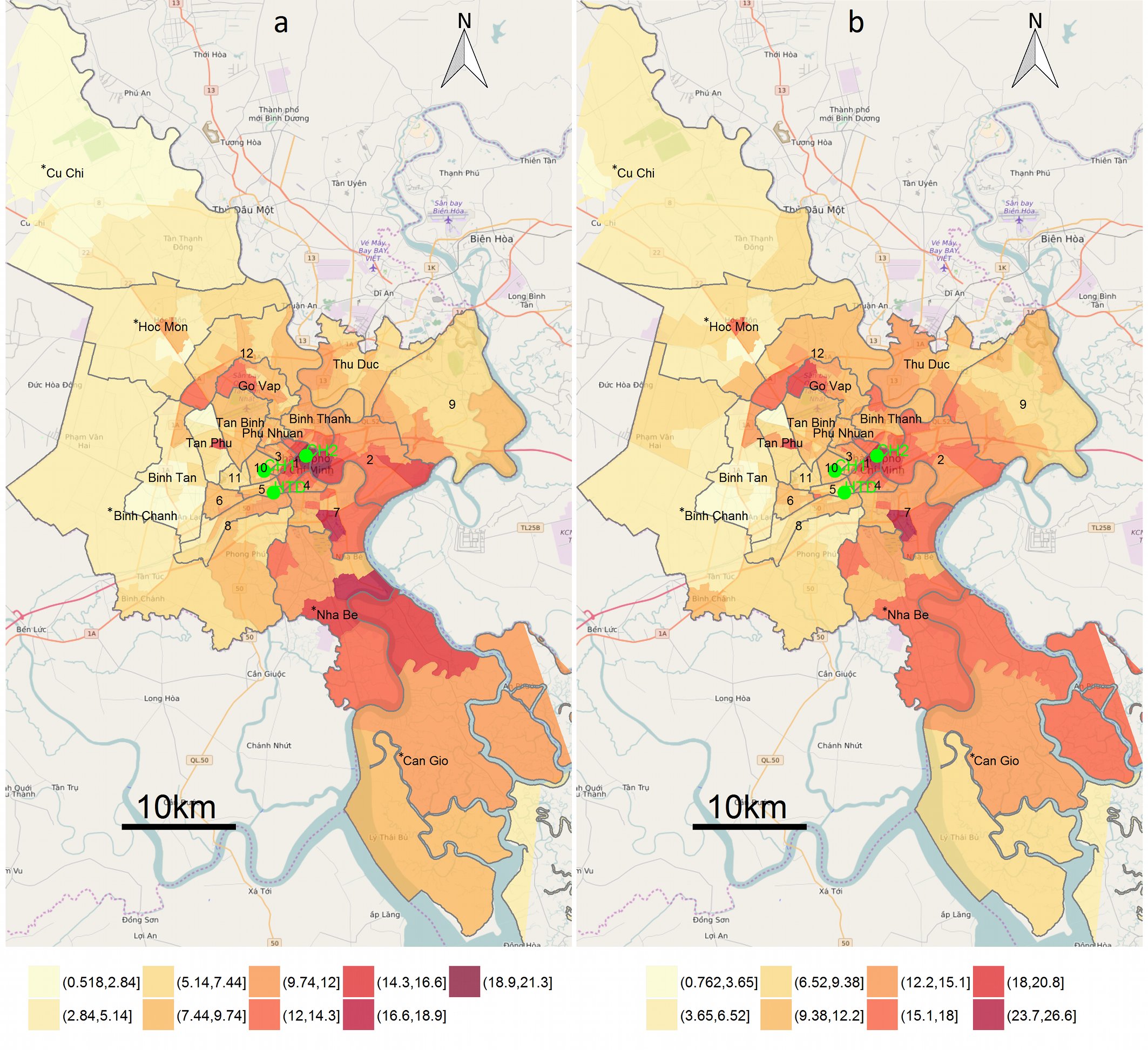

Supplement: Supplementary file 2 [file bmjopen-2017-016349supp002.jpg]

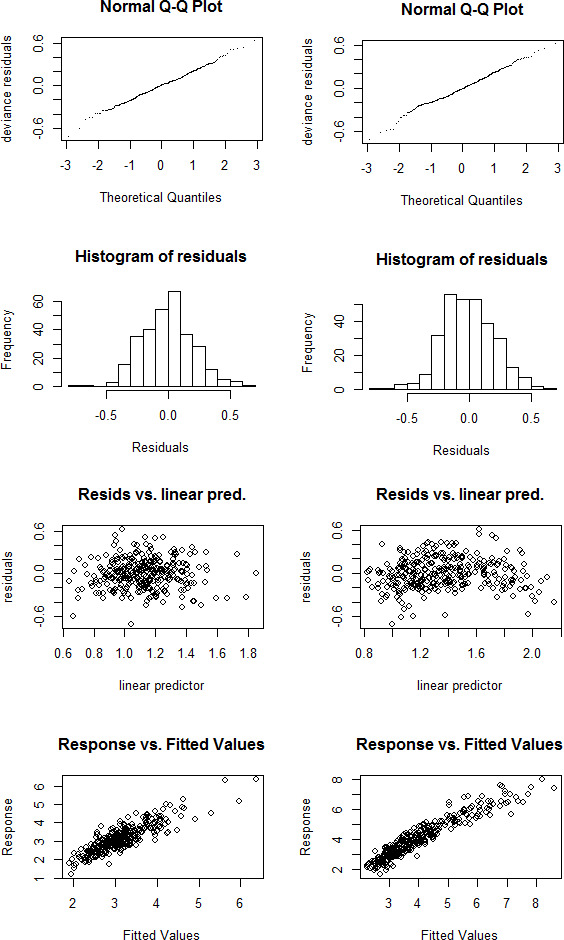

Supplement: Supplementary file 3 [file bmjopen-2017-016349supp003.jpg]

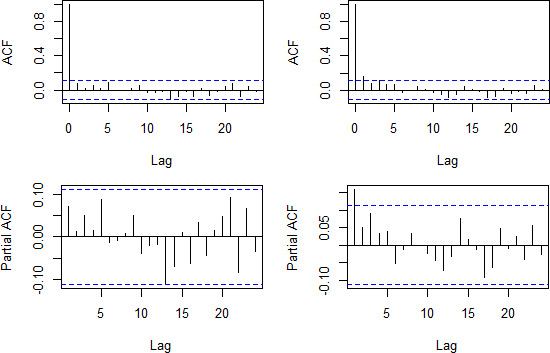

Supplement: Supplementary file 4 [file bmjopen-2017-016349supp004.jpg]
