# Supplementary material for: Retrospective analysis assessing the spatial and temporal distribution of paediatric acute respiratory tract infections in Ho Chi Minh City, Vietnam
Source: BMJ Open. 2018 Jan 21;8(1):e016349. doi: 10.1136/bmjopen-2017-016349 (PMC5780701; doi:10.1136/bmjopen-2017-016349)
Supplement: Author's manuscript [file bmjopen-2017-016349.draft_revisions.pdf]

BMJ Open is committed to open peer review. As part of this commitment we make the peer review history of every article we publish publicly available.

When an article is published we post the peer reviewers' comments and the authors' responses online. We also post the versions of the paper that were used during peer review. These are the versions that the peer review comments apply to.

The versions of the paper that follow are the versions that were submitted during the peer review process. They are not the versions of record or the final published versions. They should not be cited or distributed as the published version of this manuscript.

BMJ Open is an open access journal and the full, final, typeset and author-corrected version of record of the manuscript is available on our site with no access controls, subscription charges or pay-per-view fees (http://bmjopen.bmj.com).

If you have any questions on BMJ Open's open peer review process please email editorial.bmjopen@bmj.com

BMJ Open

A retrospective analysis assessing the spatial and temporal distribution of pediatric acute respiratory tract infections in Ho Chi Minh City, Vietnam

Journal:	*BMJ Open*
Manuscript ID	bmjopen-2017-016349
Article Type:	Research
Date Submitted by the Author:	08-Feb-2017

Complete List of Authors:	Ho, Nhan Thi; Oxford University Clinical Research Unit, Enteric infections<br>Thompson, Corinne; Oxford University Clinical Research Unit, Enteric infections<br>Nhan, Le Nguyen Thanh; Children's hospital one<br>Van, Hoang Minh Tu; Children's hospital one<br>Dung, Nguyen Thanh; The Hospital for Tropical Diseases<br>Tran My, Phuc; Oxford University Clinical Research Unit, Enteric infections<br>Quang, Vo Minh; The Hospital for Tropical Diseases<br>Minh, Ngo Ngoc Quang; The Hospital for Tropical Diseases<br>Tuan, Tran Anh; Oxford University Clinical Research Unit, Enteric infections<br>Hung, Nguyen Thanh; Children's hospital one<br>Tuan, Ha Manh ; Children's hospital 2<br>Vinh Chau, Nguyen Van; The Hospital for Tropical Diseases<br>Wolbers, Marcel; Oxford University Clinical Research Unit, Enteric infections<br>Thwaites, Guy; Oxford University Clinical Research Unit, Enteric infections<br>Choisy, Marc; Oxford University Clinical Research Unit, Enteric infections<br>Baker, Stephen; Oxford University Clinical Research Unit, Enteric infections

**Primary Subject Heading**:	Respiratory medicine
Secondary Subject Heading:	Epidemiology, Infectious diseases, Paediatrics
Keywords:	Acute respiratory infections, upper respiratory infections, lower respiratory infections, spatial distribution, temporal patterns, pediatrics

SCHOLARONE™ Manuscripts

A retrospective analysis assessing the spatial and temporal distribution of pediatric acute respiratory tract infections in Ho Chi Minh City, Vietnam

Nhan Thi Ho [1†#], Corinne Thompson [1,2,3†], Le Nguyen Thanh Nhan [4], Hoang Minh Tu Van [5], Nguyen Thanh Dung [6], Tran My Phuc [1], Vo Minh Quang [6], Ngo Ngoc Quang Minh [4], Tran Anh Tuan [4], Nguyen Thanh Hung [4], Ha Manh Tuan [5], Nguyen Van Vinh Chau [6], Marcel Wolbers [1,2], Guy E Thwaites [1,2], Marc Choisy [7] and Stephen Baker [1,2,3*]

[1] Oxford University Clinical Research Unit, Wellcome Trust Major Overseas Program, Ho Chi Minh City, Vietnam

[2] Centre for Tropical Medicine and Global Health, Nuffield Department of Medicine, University of Oxford, United Kingdom

[3] The London School of Hygiene & Tropical Medicine, United Kingdom

[4] Children's Hospital 1, Ho Chi Minh City, Vietnam

[5] Children's Hospital 2, Ho Chi Minh City, Vietnam

[6] Hospital for Tropical Diseases, Ho Chi Minh City, Vietnam

[7] Institute of Research and Development, Vietnam

† These authors contributed equally to this work.

Current institution: Columbia University Medical Center, New York, United States

* Corresponding Author: Dr. Stephen Baker, Oxford University Clinical Research Unit, 764 Vo Van Kiet, Quan 5, Ho Chi Minh City, Vietnam. Tel: +84 89241761 Fax: +84 89238904 sbaker@oucru.org

For peer review only

For peer review only - http://bmjopen.bmj.com/site/about/guidelines.xhtml

Author email adresses: Nhan Thi Ho: nth2111@cumc.columbia.edu, Corinne Thompson: corinne.thompson1@gmail.com, Le Nguyen Thanh Nhan: nhanlnt@oucru.org, Hoang Minh Tu Van: vanhmt@oucru.org, Nguyen Thanh Dung: bsdungbvbnd@gmail.com, Tran My Phuc: phuctm@oucru.org, Vo Minh Quang: bsvominhquang@gmail.com, Ngo Ngoc Quang Minh: minhnnq@yahoo.com, Tran Anh Tuan: drtat@hotmail.com, Nguyen Thanh Hung: drthanhhung@gmail.com, Ha Manh Tuan: manhtuanzu@yahoo.com, Nguyen Van Vinh Chau: chaunvv@oucru.org, Guy Thwaites: gthwaites@oucru.org, Marc Choisy: mchoisy@oucru.org, Marcel Wolbers: marcel.wolbers@gmx.ch, Stephen Baker: sbaker@oucru.org

For peer review only

For peer review only - http://bmjopen.bmj.com/site/about/guidelines.xhtml

Abstract

Background: Acute respiratory infections (ARIs) are the leading cause of morbidity and mortality in young children in developing countries. Our study examined the spatial distribution, temporal trends as well as climatic risk factors of pediatric ARIs in Vietnam.

Methods: Hospitalized pediatric (<16 years old) patients with ARIs residing in Ho Chi Minh City (HCMC) were retrieved from the 2005-2010 electronic databases of the two main Children Hospitals and the Hospital for Tropical Diseases in HCMC. Spatial mapping, time series analysis were stratified by upper respiratory infections (URIs) and lower respiratory infections (LRIs).

Results: There were 155999 pediatric patients admitted with ARIs (33% of total admissions) and 323,245 patients admitted with other conditions rather than ARIs. There were 68120 patients with URIs (14%) and 87879 patients with LRIs (19%). The most common diagnoses were acute pharyngitis (28% of all ARI), pneumonia (21%), bronchitis (18%) and bronchiolitis (16%). High ARI incidence was found mostly in districts 1, 2, 4, 7, and Nha Be. Significant increasing trend over time was found for both URIs (incidence rate ratio for 1 week increase in time (RR)=1.09, 95%CI=1.02-1.17) and LRIs (RR=1.08, 95%CI=1.006-1.16). Weekly URI incidence peaked in May-June and significantly associated with lags of URI weekly incidence and lags of weekly average humidity, rainfall and water level. Weekly LRI incidence showed a significant seasonality (p<0.0001) with a strong peak around September-October each year and significant association with lags of LRI weekly incidence and lags of weekly average temperature, rainfall and water level.

Conclusions: ARIs was the leading cause of childhood hospitalization in HCMC, Vietnam. The incidence of ARIs was higher in rainy season and higher in districts 1, 2, 4, 7 and Nha Be. These results may guide the health authorities in where and when to effectively allocate resources for the prevention and control of ARIs.

For peer review only - http://bmjopen.bmj.com/site/about/guidelines.xhtml

Strengths and limitations of the study

- This study was the first examining the spatial distribution, temporal patterns and climatic risk factors of acute respiratory infections (ARIs) in Vietnam
- Using routinely collected electronic hospital databases, a large number of ARI admissions over 6 years period was retrieved based on ICD10 codes and analyzed in comparison with all admissions due to other conditions rather than ARIs.
- Analyses were stratified by two clinically differentiated groups: upper respiratory infections (URIs) and lower respiratory infections (LRIs) to address the potential lack of correctness or lack of details of the ICD10 codes in the databases.
- Spatial distribution of ARIs found from hospital databases might influenced by hospital referral pattern.
- There was a lack of information on the specific pathogens of ARIs in the databases

Key words

Acute respiratory infections, upper respiratory infections, lower respiratory infections, spatial distribution, temporal patterns, pediatrics

For peer review only - http://bmjopen.bmj.com/site/about/guidelines.xhtml

For peer review only

Introduction

Acute respiratory tract infections (ARIs) are composed of upper respiratory tract infections (URIs) and lower respiratory infections (LRIs). URIs are associated with infections at or above the larynx, are most commonly induced by viruses and include rhinitis (the common cold), sinusitis, ear infections, acute pharyngitis, epiglottitis, and laryngitis. LRIs are comprised of bacterial or viral infections occurring below the larynx and encompass bronchitis, bronchiolitis, and pneumonia [1]. ARIs are the leading cause of morbidity and mortality in young children [2–4], accounting for more than 18% of all childhood deaths globally [5]. In 2010, there were approximately 120 million episodes and 1.3 million deaths associated with pneumonia only worldwide, the vast majority of these occurred in Southeast Asia and sub-Saharan Africa [5,6]. In industrializing regions, ARIs in children aged less than five years are generally more severe than in older children and adults and result in a higher case-fatality than in industrialized countries [1,7]. This disparity in mortality rate is multifactorial and associated with differing etiological agents, available therapies, exposure frequencies, and host immunity.

In Ho Chi Minh City (HCMC), Vietnam, ARIs are the most common cause of childhood hospitalizations [8–10]. The common identified pathogens of hospitalized ARI in HCMC include respiratory syncytial virus (RSV), rhinovirus, coronavirus, influenza, and bocavirus [8–10] in addition to the bacterial pathogens *Streptococcus pneumoniae* and *Haemophilus influenzae* type b (Hib) [11,12]. With limited access to costly vaccines for bacterial etiologies and a lack of vaccines for viral causes of ARIs, a greater understanding of the risk factors and sub-populations at risk of ARIs in HCMC need to focus on existing prevention measures such as the encouragement of breastfeeding, hand washing with soap and a reduction of household air pollution [2].

There is evidence to suggest that the incidence of ARIs is associated with specific spatial, meteorological and socio-demographic risk factors [13–16]. However, specific temporal and climatic risk factors for both URIs and LRIs in HCMC have not been investigated to date. Here we aimed to

For peer review only - http://bmjopen.bmj.com/site/about/guidelines.xhtml

examine the spatial distribution, temporal patterns, and climatic risk factors for hospitalized pediatric ARIs in HCMC to generate a better understanding of the factors that may influence ARIs and in order to guide healthcare resources for this common pediatric disease syndrome.

Methods

Data sources

Data for this study was derived from the electronic hospital databases of all pediatric (<16 years) inpatient admissions to three large tertiary hospitals located in central HCMC: Children's Hospital 1 (CH1), Children's Hospital 2 (CH2) and the Hospital for Tropical Diseases (HTD). The data of the two children hospitals were collected between 2005 and 2010 (inclusive) while the data from HTD were only available from 2008 to 2010.

ARIs are caused by a range of differing pathogens and may have different spatiotemporal patterns and associations with climate and other covariates. Therefore, we allocated respiratory infections into two major groups based on the International Classification of Disease version 10 (ICD10) codes version 2016 outlined by the World Health Organization (WHO): 1) URIs were amalgamated under the ICD10 codes J00:J06 and J9:J11 for influenza like illnesses and other acute upper respiratory infections and 2) LRIs, which incorporated the ICD10 codes J12:J18 and J20:J22 for pneumonia and other acute lower respiratory infections. For the purposes of this study the data from patients with the selected ICD10 codes who resided in HCMC were extracted and analyzed. Each patient record included data regarding age, sex, date of admission and discharge, ICD10 code at discharge, and residential address

HCMC is subdivided into 24 districts (land areas ranging from 4.18 km^2- 704.22 km^2), which are themselves subdivided in 322 smaller administrative wards. There are 5 rural districts (population density ranging from 100 persons/km2 to 3,326 persons/km2) and 19 urban districts (population density ranging from 2,360 persons/km2 to 45,582 persons/km2). There are two main seasons in HCMC: dry season

For peer review only - http://bmjopen.bmj.com/site/about/guidelines.xhtml

(from December to April) and rainy season (from May to November). Population and district characteristic data (urban vs. rural districts, district population density, district economic indicators (expenditure of district budget, revenues of district budget), district agricultural indicators (number of pigs in herds > 2 months of age by district, % area of district used as rice paddy), district education indicator (number of primary and secondary schools per district)) were obtained from the HCMC Statistical Office [17]. As population figures for those aged <16 years per ward or district was not obtainable, the total population was used to estimate disease incidence. Patient addresses in HCMC were geocoded to districts and wards. City level weekly average climate data during 2005–2010 were obtained from the Ministry of Natural Resources and Environment of Vietnam including relative humidity (median=76.5%, range=61.7% to 88%), temperature (median=28.1°C, range=24.4°C to 31.8°C), rainfall (median=24.4mm, range=0mm to 230.7mm) and water level of the Dong Dien River (median=4.93cm, range= -34.43cm to 49.43cm) [18].

Spatial mapping

To reduce variation in the variance of raw incidence estimates due to the variation in population size, ward incidence rates (number of ARI cases per 1,000 ward total population for the whole study period) were smoothed using local empirical Bayes estimates for rates reduced to a neighborhood mean. The neighborhoods were selected using a the neighborhood list based on wards with contiguous boundaries (R package spdep version 0.5-88) [19]. Kulldorff and Nagarwalla's method over the centroids was be used to scan for clusters of diseases [20].

Poisson generalized linear model was used to explore the relationship between district CNSI incidence and district characteristics. Confidence intervals and tests were based on robust standard errors to control for mild violation of the distributional assumption that the variance equals the mean [21].

For peer review only - http://bmjopen.bmj.com/site/about/guidelines.xhtml

Time series analysis

The time series of citywide ARI weekly incidences (number of cases per 1,000 population) were examined for seasonality, time trends and associations with climatic covariates by a Poisson generalized additive mixed model (GAMM) implemented in the R package mgcv version 1.8-7 [22,23]. The seasonal cycles and time trends of weekly ARI incidence were evaluated by the basic GAMM models containing the week of year (Ws=1 to 52)) as cyclic cubic regression splines and the week of the whole study period (Wt=1 to 312)) as cubic regression spline. As weekly ARI incidence may be associated with the ARI incidence and climatic condition of the previous weeks, in addition to the elements in the basic model, the full GAMM model also contained lags of ARI weekly incidence up to eight weeks, concurrent climatic condition and lags of climatic condition up to 8 weeks. The performance of the GAMM models was evaluated by adjusted R-squared and model diagnostic plots. Temporal correlation of the model residuals was evaluated by autocorrelation function (ACF) and partial autocorrelation function (PACF). Spatial mapping and statistical analyses were stratified by URIs and LRIs and all were performed in R version 3.2.2 [24].

Results

Basic features of acute respiratory infections in the pediatric population of Ho Chi Minh City

From 2005 to 2010, there were 155,999 children (<16 years old) residing in HCMC admitted to the three study hospitals for ARIs (33% of all admissions); 323,245 patients were admitted due to other conditions rather than ARIs. There were 68,120 patients with ICD10 codes synonymous with acute URIs (14% of all admissions) and 87,879 patients with acute LRIs (19% of all admissions). The proportion of males (62%) and females (38%) was comparable between URIs and LRIs. The median age of the patients was 1.5 years (interquartile range [IQR]: 0.7-2.9 years). However, patients with LRIs were significantly younger than those with URIs (median age in years (IQR) = 1.1 (0.5, 2.1) vs. 2.1 (1.1, 3.9)), Wilcoxon's test *p*<0.0001) and LRI patients were hospitalized significantly longer than those with URIs (median days of hospitalization (IQR) = 6.0 (4.0, 9.0) vs. 4.0 (2.0, 5.0), Wilcoxon's test *p*<0.0001).

For peer review only - http://bmjopen.bmj.com/site/about/guidelines.xhtml

For peer review only

Overall, the most common diagnoses were acute pharyngitis (28% of all ARIs), pneumonia (21% of all ARIs), bronchitis (18% of all ARIs) and bronchiolitis (16% of all ARIs). Among the URI patients, the majority of patients were diagnosed with acute pharyngitis (63% of all URIs), acute upper respiratory infections of multiple and unspecified sites (14%), acute tonsillitis (8%), acute laryngitis and tracheitis (6%), and acute nasopharyngitis (6%). Among LRI patients, patients were diagnosed with pneumonia with an unspecified organism (37% of all LRIs), acute bronchitis (32%), acute bronchiolitis (29%), and bacterial pneumonia not elsewhere classified (2%) (Table 1).

The spatial distribution of pediatric acute respiratory infections in Ho Chi Minh City

The raw ward-level incidence rates of ARIs for the whole study period ranged from zero to 50 per 1,000 total population (median (IQR) = 18.8 (14.2, 23.5)) and the smoothed ward level empirical Bayesian estimated ARI incidence rates ranged from 1.2 to 48.8 ARI cases per 1,000 population (median (IQR) = 18.6 (14.7, 23.7)). High incidence rates (>30 cases/1,000 population) of ARI were observed not only in the wards surrounding the three study hospitals (wards in districts 1 and 4 in central HCMC) but also in multiple wards located outside the central region of the city, specifically wards located in districts 2, 7, and Nha Be (observed vs. expected incidence rate ratio>1.4, p<0.0001 with Kulldorff and Nagarwalla's scan) (Figure 1a). Notably, the spatial distribution of ARIs and diseases other than ARI were comparable (Figure 1a and 1b). We observed an increase over time in ward incidence of ARIs in the majority of the wards with the most apparent high incidences in wards of in the southern orientated districts of 7 and Nha Be (Figure S1). We additionally found the spatial distribution of URIs and LRIs was comparable (Figure S2).

The district level incidence over the entire study period of ARIs was significantly lower in the districts located further away from the hospitals (RR for 2 times increase in distance to hospital = 0.88; 95% CI =

For peer review only - http://bmjopen.bmj.com/site/about/guidelines.xhtml

0.80, 0.96) and for rural districts (RR for urban vs. rural districts= 1.54; 95% CI = 1.10, 2.15). Other district characteristics were not significantly associated with district ARI incidence.

Temporal patterns and climatic risk factors of pediatric acute respiratory infections

The mean of weekly incidence of URIs for the study period was 3.12/1,000 population. Weekly URI incidence showed a relatively small increase through time, with a difference in mean weekly URI incidence at the end and the start of the study period of 0.86 patients per 1,000 population. The basic Poisson GAMM model indicated that URIs admissions in the study hospitals exhibited a significant temporal increasing trend (p<0.0013) and seasonality (p<0.0001) with a peak in May to June and a smaller peak in December to January (Figure 2a).

The mean of weekly LRI incidence for the study period was 4.02/1,000 population. There was a significant increase of weekly LRI incidence over time (p=0.0037) with a substantial difference in the mean weekly LRI incidence at the end vs. the start of the study period of 1.54 patients/1,000 population. The seasonal cycle of LRIs (p<0.0001) was more obvious than that of URIs with a single strong and wide peak annually in the months of September and October (Figure 2b).

In a univariate analysis with Poisson generalized linear model, we found that weekly URI incidence was significantly associated with concurrent weekly average temperature (RR for 1 degree increase of temperature=1.048, 95%CI=1.030-1.066) and humidity (RR for 1% increase in relative humidity=1.006, 95%CI= 1.001-1.011). Further, weekly LRI incidence was significantly associated with concurrent weekly average humidity (RR=1.030, 95%CI=1.024-1.036) and rainfall (RR for 1mm increase in rainfall= 1.019, 95%CI=1.014-1.024) (Figure 3). However, after controlling for seasonality, time trend, lags of weekly incidence, concurrent and lags of other climatic covariates in the full GAMM model, the weekly URI incidence was significantly associated with one and two week lags in URI incidence (RR=1.202, 95%CI=1.159-1.245 and RR=1.063, 95%CI=1.019-1.109, respectively), one and three week

For peer review only - http://bmjopen.bmj.com/site/about/guidelines.xhtml

lags in weekly average humidity (RR=1.009, 95%CI=1.003-1.014 and RR=0.990, 95%CI=0.984-0.996, respectively), a three week lag in weekly average rainfall (RR=1.006, 95%CI=1.003-1.009) and a four week lag in weekly average river water level (RR for 1 cm increase in water level=1.003, 95%CI=1.002-1.005).

After adjusting for other factors in the full GAMM model, weekly LRI incidence was significantly associated with a one and two week lags in LRI incidence (RR=1.173, 95%CI=1.138-1.208 and RR=1.053, 95%CI=1.015-1.092 respectively), a one week lag in weekly average temperature (RR=0.976, 95%CI=0.953-0.998), a one week lag in weekly average rainfall (RR=1.004, 95%CI=1.001-1.007) and a one week lag in weekly average water level (RR=0.998, 95%CI=0.997-1.000) (Table 2). The time trends remained significant for both URIs (p=0.0096) and LRIs (p=0.0345) while only LRIs exhibited significant seasonality (p<0.0001) in the full GAMM models. The full GAMM models fitted the data well for both URIs (adjusted R squared =0.72) and LRIs (adjusted R squared =0.88) (Figure 2 and Figure S3). Temporal correlation of weekly incidence was well controlled for URIs and also relatively well controlled for LRIs in the full GAMM models (Figure S4).

Discussion

This study is the first examining the spatial distribution, temporal patterns and climatic risk factors of ARIs in Vietnam. Our data show that ARIs accounted for 1/3 of all hospital admissions over the study period and were the most common cause of hospitalized disease in children in HCMC. The spatial distribution of ARIs was comparable to the spatial distribution of other diseases rather than ARIs admitted to our three study hospitals in HCMC, and no specific distribution was observed for ARIs as compared to other diseases rather than ARIs. High incidence of ARIs was found not only in districts close to the study hospitals (districts 1, 4) but also in districts 2, 7 and Nha Be, which are located away from three study hospitals. This finding may be a result of urbanization, including better transportation to the three study hospitals or better economic status in these districts during the study period while local

For peer review only

For peer review only - http://bmjopen.bmj.com/site/about/guidelines.xhtml

healthcare facilities might not have grown proportionally to meet the increasing healthcare need in these areas. The increase in hospitalized ARI incidence over time that we observed could also be explained by better healthcare awareness in the population, through better access to tertiary hospitals or a real increase in the incidence of ARIs. The citywide increase of ARI incidence, especially LRI, could also be attributed to the increase of pollution. Pollution was shown to be associated with an increase in LRI incidence by the study of the Collaborative Working Group on Air Pollution, Poverty, and Health in HCMC [25].

The weekly incidence of both URIs and LRIs showed significant association with 1 and 2 week lags of incidence indicating that temporal correlation plays an important role in URI and LRI incidence. Furthermore, we additionally noted a difference in seasonal patterns of URIs (two small peaks a year) and LRIs (one strong peak a year) and a difference in association patterns with climatic factors of URIs and LRIs. These findings suggest that climatic factors may differentially influence the pathogens causing URIs and LRIs in HCMC. In addition, the fact that a sharp increase of LRI incidence was observed soon after the peak of URI (May-June) indicate that URIs and LRIs may share some common epidemiological features or LRIs were associated with URI complications or even the pathogens of LRIs infected URI patients. A study conducted at Children Hospital 2 (one of the three study hospitals) investigating viral pathogens associated with ARIs found that the peak of RSV infections occurred in rainy season (May-November in HCMC). This period includes the peak of LRI months and the stronger peak of URIs [10]. Furthermore, RSV is also thought to predispose infected hosts to bacterial infection [26]. Therefore, RSV is likely to be an important pathogen with respect to seasonality and the peaks of URIs and LRIs in this location. As a consequence, appropriate treatment and transmission control for URIs may impact on the incidence of later hospitalization due to LRI. As the peaks of hospitalization for LRI are around September-October, resources for pediatric healthcare should be prepared and local healthcare facilities should be enhanced to share the increase in hospitalization caused by LRIs at tertiary hospitals during this period.

For peer review only - http://bmjopen.bmj.com/site/about/guidelines.xhtml

For peer review only

310 Our study has limitations as it was derived from routine electronic hospital databases. First, it might not be generalizable for all outpatients, especially for URIs, as many of patients with URI may not be admitted to hospital. The findings for LRIs may be more generalizable as LRIs are more severe than URIs and more likely to be hospitalized. Second, the databases contained limited information with general ICD10 codes and no specific diagnostic information regarding the infecting pathogens. Different pathogens may cause differing URI and LRI disease presentation and different pathogens may be influenced differently by climatic, environmental, population factors as well as host immunity. Therefore, prospective studies investigating different groups of pathogens for ARIs are necessary.

Conclusion

We found that ARIs accounted for 1/3 of total hospital admission and were the leading cause of hospitalization in children in HCMC, Vietnam. The spatial distribution of hospitalized ARIs was similar to general spatial distribution of other hospitalized diseases with high incidences in the districts surrounding the locations of the three study hospitals and also in districts 2, 7 and Nha Be. The seasonality of ARIs, with strong peak in rainy season, might be explained by the association of ARI incidence with climatic factors (temperature, humidity and rainfall) and the seasonality of RSV infection. The increase of LRI incidence soon after the peak of URIs suggests that URIs and LRIs share common epidemiological pathways or that LRIs are complications of URIs. Understanding the spatial distribution, temporal patterns and climatic risk factors of ARIs should aid health authorities in outbreak preparation and assist in allocating resources for the prevention and control of the diseases, especially in settings where vaccines for viral and bacterial pathogens of ARIs are not routinely available.

Acknowledgements

The authors are grateful to the staffs of Children's Hospital 1, Children's Hospital 2 and The Hospital for Tropical Diseases for providing the raw databases used in this study.

For peer review only - http://bmjopen.bmj.com/site/about/guidelines.xhtml

Footnotes

Contributors

This study was conceptualized by NTH CT, MC and SB. Data was obtained by NTH, CT HTH, CT, LNTN, HMTV, NTD, TMP, VMQ, NNQM, TAT, NTH, HMT and NVVC. Data was analyzed by NTH, CT, MW, MC. NTH, CT and SB. NTH, CT and SB wrote the manuscript with input from all authors. HTH, CT, LNTN, HMTV, NTD, TMP, VMQ, NNQM, TAT, NTH, HMT, NVVC, MW, GET, MC, SB read and approved the final version of the manuscript.

Funding

This work was supported by the Wellcome Trust Vizions strategic award (WT1093824). SB is a Sir Henry Dale Fellow, jointly funded by the Wellcome Trust and the Royal Society (100087/Z/12/Z). NTH is currently funded by Mervyn Susser's fellowship at Columbia University Medical Center, US. The funders had no role in study design, data collection, data analysis, data interpretation, writing of the report, or decision to submit the article for publication.

Competing interests

We have read and understood BMJ policy on declaration of interests and declare that we have no competing interests.

Ethical approval

Ethical approval was granted from all of the three study hospitals: Children Hospital 1, Children Hospital 2 and the Hospital for Tropical Diseases.

Data sharing statement

Data will be available upon reasonable request to corresponding author.

For peer review only

References

1. Simoes EAF, Cherian T, Chow J, Shahid-Salles SARL, John TJ. Acute Respiratory Infections in Children. Disease Control Priorities in Developing Countries. 2nd ed. Washington DC: World Bank; 2006.
2. WHO/UNICEF Ending Preventable Child Deaths from Pneumonia and Diarrhoea by 2025: The Integrated Global Action Plan for Pneumonia and Diarrhoea (GAPPD). Geneva; 2013.
3. Lozano R, Naghavi M, Foreman K, Lim S, Shibuya K, Aboyans V, et al. Global and regional mortality from 235 causes of death for 20 age groups in 1990 and 2010: A systematic analysis for the Global Burden of Disease Study 2010. Lancet. 2012;380: 2095–2128. doi:10.1016/S0140-6736(12)61728-0
4. Williams BG, Gouws E, Boschi-Pinto C, Bryce J, Dye C. Estimates of world-wide distribution of child deaths from acute respiratory infections. Lancet Infectious Diseases. 2002. pp. 25–32. doi:10.1016/S1473-3099(01)00170-0
5. Liu L, Johnson HL, Cousens S, Perin J, Scott S, Lawn JE, et al. Global, regional, and national causes of child mortality: An updated systematic analysis for 2010 with time trends since 2000. Lancet. 2012;379: 2151–2161. doi:10.1016/S0140-6736(12)60560-1
6. Fischer Walker CL, Rudan I, Liu L, Nair H, Theodoratou E, Bhutta ZA, et al. Global burden of childhood pneumonia and diarrhoea. Lancet. 2013;381: 1405–1416. doi:10.1016/S0140-6736(13)60222-6
7. Global Burden of Disease Pediatrics Collaboration, Kyu HH, Pinho C, Wagner JA, Brown JC, Bertozzi-Villa A, et al. Global and National Burden of Diseases and Injuries Among Children and Adolescents Between 1990 and 2013: Findings From the Global Burden of Disease 2013 Study. JAMA Pediatr. 2016;98121: 1–21. doi:10.1001/jamapediatrics.2015.4276
8. Anders KL, Nguyen HL, Nguyen NM, Thuy NT Van, Van NTH, Hieu NT, et al. Epidemiology and virology of acute respiratory infections during the first year of life: a birth cohort study in Vietnam. Pediatr Infect Dis J. 2015;34: 361–370.

For peer review only - http://bmjopen.bmj.com/site/about/guidelines.xhtml

9. Do AHL, van Doorn HR, Nghiem MN, Bryant JE, Hoang TH thi, Do QH, et al. Viral etiologies of acute respiratory infections among hospitalized vietnamese children in Ho Chi Minh City, 2004-2008. PLoS One. 2011;6. doi:10.1371/journal.pone.0018176

10. Tran DN, Trinh QD, Pham NTK, Vu MP, Ha MT, Nguyen TQN, et al. Clinical and epidemiological characteristics of acute respiratory virus infections in Vietnamese children. Epidemiol Infect. 2015; 1–10. doi:10.1017/S095026881500134X

11. Jauneikaite E, Jefferies JM, Hibberd ML, Clarke SC. Prevalence of Streptococcus pneumoniae serotypes causing invasive and non-invasive disease in South East Asia: A review. Vaccine. 2012;30: 3503–3514. doi:10.1016/j.vaccine.2012.03.066

12. Nyambat B, Dang DA, Nguyen HA, Mai TQ, Rani M, Slack MPE, et al. Rapid assessment of Hib disease burden in Vietnam. BMC Public Health. 2011;11: 260. doi:10.1186/1471-2458-11-260

13. du Prel J-B, Puppe W, Gröndahl B, Knuf M, Weigl JAI, Schaaff F, et al. Are meteorological parameters associated with acute respiratory tract infections? Clin Infect Dis. 2009;49: 861–868. doi:10.1086/605435

14. Chen Z, Ji W, Wang Y, Yan Y, Zhu H, Shao X, et al. Epidemiology and associations with climatic conditions of Mycoplasma pneumoniae and Chlamydophila pneumoniae infections among Chinese children hospitalized with acute respiratory infections. Ital J Pediatr. 2013;39: 34. doi:10.1186/1824-7288-39-34

15. Welliver R. The relationship of meteorological conditions to the epidemic activity of respiratory syncytial virus. Paediatr Respir Rev. 2009;10: 6–8. doi:10.1016/S1526-0542(09)70004-1

16. Cardoso MRA, Cousens SN, de Góes Siqueira LF, Alves FM, D'Angelo LA V. Crowding: risk factor or protective factor for lower respiratory disease in young children? BMC Public Health. 2004;4: 19. doi:10.1186/1471-2458-4-19

17. Statistical Office in Ho Chi Minh City. Statistical Yearbook of Ho Chi Minh City 2011. Ho Chi Minh City: Ho Chi Minh City Statistical Office; 2012.

18. Loc PD. Ministry of Natural Resources and Environment of the Socialist Republic of Vietnam. Ha

For peer review only - http://bmjopen.bmj.com/site/about/guidelines.xhtml

Noi; 2013.

19. Bivand R, Hauke J, Kossowski T. Computing the jacobian in gaussian spatial autoregressive models: An illustrated comparison of available methods. Geogr Anal. 2013;45: 150–179. doi:10.1111/gean.12008

20. Kulldorff M, Nagarwalla N. Spatial disease clusters: detection and inference. Stat Med. 1995;14: 799–810. doi:10.1002/sim.4780140809

21. Cameron AC, Trivedi PK. Microeconometrics using Stata. Stata Press books. 2009;5: 706. doi:10.1016/S0304-4076(00)00050-6

22. Lin X, Zhang D. Inference in generalized additive mixed models by using smoothing splines. J R Stat Soc Ser B. 1999;61: 381–400.

23. Wood SN. Generalized additive models: an introduction with R. Wiley Interdiscip Rev Comput. 2006;16.

24. R Core Team. R: A Language and Environment for Statistical Computing. R Foundation for Statistical Computing Vienna Austria. 2014. p. {ISBN} 3-900051-07-0. doi:10.1017/CBO9781107415324.004

25. HEI Collaborative Working Group on Air Pollution and Health in Ho Chi Minh City P, Le TG, Ngo L, Mehta S, Do VD, Thach TQ, et al. Effects of short-term exposure to air pollution on hospital admissions of young children for acute lower respiratory infections in Ho Chi Minh City, Vietnam. Res Rep Health Eff Inst. 2012; 5–83.

26. Beadling C, Slifka MK. How do viral infections predispose patients to bacterial infections? Curr Opin Infect Dis. 2004;17: 185–191. doi:10.1097/00001432-200406000-00003

For peer review only - http://bmjopen.bmj.com/site/about/guidelines.xhtml

437 **Tables**

438 **Table 1. Characteristics and ICD10 diagnoses at admission for all ARI patients, those with upper**
439 **respiratory infection (URI) and those with lower respiratory infection (LRI)**

Characteristic	All ARI patients (N=155999)	URI (N=68120)	LRI (N=87879)	p-value
Male sex[a]	96930/155997 (62%)	42251/68119 (62%)	54679/87878 (62%)	0.43*
Age (year)[b]	1.5(0.7,2.9)	2.1(1.1,3.9)	1.1(0.5,2.1)	<0.0001#
Days in hospital[c]	5.0(3.0,7.0)	4.0(2.0,5.0)	6.0(4.0,9.0)	<0.0001#
**ICD10 code at discharge**				
Acute bronchiolitis (J21)	25123/155999 (16%)	0/68120 (0%)	25123/87879 (29%)	
Acute bronchitis (J20)	28202/155999 (18%)	0/68120 (0%)	28202/87879 (32%)	
Acute laryngitis & tracheitis (J04)	4323/155999 (3%)	4323/68120 (6%)	0/87879 (0%)	
Acute nasopharyngitis (J00)	4049/155999 (3%)	4049/68120 (6%)	0/87879 (0%)	
Acute obstructive laryngitis & epiglottitis (J05)	9/155999 (0%)	9/68120 (0%)	0/87879 (0%)	
Acute pharyngitis (J02)	43148/155999 (28%)	43148/68120 (63%)	0/87879 (0%)	
Acute sinusitis (J01)	592/155999 (0%)	592/68120 (1%)	0/87879 (0%)	
Acute tonsillitis (J03)	5123/155999 (3%)	5123/68120 (8%)	0/87879 (0%)	
Acute upper respiratory infections of multiple & unspecified sites (J06)	9300/155999 (6%)	9300/68120 (14%)	0/87879 (0%)	
Bacterial pneumonia, not elsewhere classified (J15)	2016/155999 (1%)	0/68120 (0%)	2016/87879 (2%)	
Influenza due to other identified virus (J10)	381/155999 (0%)	381/68120 (1%)	0/87879 (0%)	
Influenza, virus not identified (J11)	1195/155999 (1%)	1195/68120 (2%)	0/87879 (0%)	
Pneumonia due to *Haemophilus influenza* (J14)	11/155999 (0%)	0/68120 (0%)	11/87879 (0%)	
Pneumonia due to other infectious organisms, not elsewhere classified (J16)	26/155999 (0%)	0/68120 (0%)	26/87879 (0%)	
Pneumonia due to *Streptococcus pneumonia* (J13)	16/155999 (0%)	0/68120 (0%)	16/87879 (0%)	
Pneumonia in diseases classified elsewhere (J17)	1/155999 (0%)	0/68120 (0%)	1/87879 (0%)	
Pneumonia, organism unspecified (J18)	32371/155999 (21%)	0/68120 (0%)	32371/87879 (37%)	
Unspecified acute lower respiratory infection (J22)	7/155999 (0%)	0/68120 (0%)	7/87879 (0%)	
Viral pneumonia, not elsewhere classified (J12)	106/155999 (0%)	0/68120 (0%)	106/87879 (0%)	

440 a: data from 155997 patients; b: data from 147420 patients; c: data from 96362 patients.

For peer review only - http://bmjopen.bmj.com/site/about/guidelines.xhtml

441 *From Chi-square test comparing URIs vs. LRIs; #From Wilcoxon's rank sum test comparing URIs vs.
442 LRIs.
443

For peer review only

For peer review only - http://bmjopen.bmj.com/site/about/guidelines.xhtml

Table 2. Results of the full generalized additive mixed models (GAMM) for upper respiratory infection (URI) and lower respiratory infection (LRI).

	URI				LRI			
	**RR**	**LL**	**UL**	**P value**	**RR**	**LL**	**UL**	**P value**
Intercept	0.340	0.133	0.869	0.0251	1.421	0.381	5.303	0.6013
Lag 1 week incidence	1.202	1.159	1.245	0.0000	1.173	1.138	1.208	0.0000
Lag 2 week incidence	1.063	1.019	1.109	0.0048	1.053	1.015	1.092	0.0060
Lag 3 week incidence	0.999	0.957	1.042	0.9470	0.987	0.951	1.024	0.4861
Lag 4 week incidence	0.974	0.934	1.017	0.2328	0.998	0.962	1.035	0.9180
Temperature (°C)	1.017	0.996	1.040	0.1178	1.006	0.985	1.028	0.5748
Lag 1 week temperature	0.990	0.966	1.015	0.4480	0.976	0.953	0.998	0.0348
Lag 2 week temperature	1.019	0.994	1.045	0.1389	1.007	0.984	1.030	0.5620
Lag 3 week temperature	1.010	0.985	1.035	0.4470	1.019	0.995	1.043	0.1186
Lag 4week temperature	1.012	0.990	1.035	0.2709	1.015	0.993	1.038	0.1722
Humidity (%)	0.998	0.993	1.004	0.5619	0.998	0.993	1.004	0.5506
Lag 1 week humidity	1.009	1.003	1.014	0.0051	0.997	0.991	1.002	0.2716
Lag 2 week humidity	1.002	0.996	1.007	0.6000	1.001	0.995	1.006	0.7603
Lag 3 week humidity	0.990	0.984	0.996	0.0008	1.000	0.995	1.006	0.9237
Lag 4 week humidity	1.005	0.999	1.010	0.1024	1.000	0.994	1.005	0.8610
Rainfall (mm)	1.000	0.997	1.003	0.9670	1.001	0.998	1.004	0.5615
Lag 1 week rainfall	1.001	0.997	1.004	0.7145	1.004	1.001	1.007	0.0073
Lag 2 week rainfall	1.002	0.998	1.005	0.2979	1.000	0.998	1.003	0.7548
Lag 3 week rainfall	1.006	1.003	1.009	0.0005	1.001	0.998	1.004	0.4927
Lag 4 week rainfall	0.997	0.994	1.001	0.1163	0.999	0.997	1.002	0.6949
Water level (cm)	0.999	0.997	1.000	0.1519	0.999	0.997	1.000	0.0751
Lag 1 week water level	1.001	0.999	1.002	0.2120	0.998	0.997	1.000	0.0111
Lag 2 week water level	0.999	0.998	1.001	0.2488	1.000	0.998	1.001	0.7821
Lag 3 week water level	1.000	0.998	1.001	0.7223	1.000	0.999	1.002	0.9576
Lag 4 week water level	1.003	1.002	1.005	0.0000	1.001	1.000	1.003	0.1473
Time trend (week)	1.092	1.022	1.166	0.0096	1.080	1.006	1.159	0.0345

Results of fixed effects (with only the results of lags up to 4 weeks) are shown.

P values for seasonal cycles from the full GAMM of URI=0.5177 and LRI<0.0001 respectively.

Adjusted R-squared of the full GAMM for URI=0.72 and LRI=0.88 respectively.

For peer review only

For peer review only - http://bmjopen.bmj.com/site/about/guidelines.xhtml

449 RR: rate ratio; LL and UL: lower and upper limit of 95% confidence interval.

For peer review only

For peer review only - http://bmjopen.bmj.com/site/about/guidelines.xhtml

Figure legends

Figure 1. Maps for empirical Bayesian estimated incidence rates of acute respiratory infection (ARI) and other diseases at ward level.

a) Ward level empirical Bayesian estimated incidence rates (EBR) per 1000 population for ARI with locations of the 3 study hospitals in green, b) ward level EBR for other diseases rather than ARI. CH1: Children Hospital 1, CH2: Children Hospital 2; HTD: Hospital for Tropical Diseases. District names and district boundaries (grey) are shown. Rural districts are marked with *.

Figure 2. GAMM plots of city wide weekly incidence time series for URI and LRI

Upper panels (a): smoothed seasonal cycles (red line), time trend (blue line) from the basic Poisson generalized additive mixed model (GAMM), fitted values (green line) from the full Poisson GAMM model and observed values (black line) of citywide weekly incidence (number of cases per 1000 population) of upper respiratory infections (URI).

Lower panels (b): smoothed seasonal cycles (red line), time trend (blue line) from the basic Poisson GAMM model and fitted values (green line) from the full Poisson GAMM model and observed values (black line) of citywide weekly incidence of lower respiratory infections (LRI).

Figure 3. Time series of city wide weekly incidence of URI and LRI vs. weekly climatic covariates

URI: upper respiratory infection; LRI: lower respiratory infection.

Left panel: time series of weekly URI and LRI incidence and weekly average climatic covariates.

Right panel: Scatter plots with fitted lines and 95% confidence interval from univariate Poisson generalized linear model for weekly URI or LRI incidence vs. each climatic covariate.

For peer review only - http://bmjopen.bmj.com/site/about/guidelines.xhtml

For peer review only

Supplementary Figures

Figure S1. Maps for empirical Bayesian estimated incidence rates (EBR) of acute respiratory infection (ARI) by year at ward level

District labels are shown. 1-12: district 1-12; 13-24: district Binh Chanh, Binh Tan, Binh Thanh, Can Gio, Cu Chi, Go Vap, Hoc Mon, Nha Be, Phu Nhuan, Tan Binh, Tan Phu, Thu Duc respectively. Locations of study hospitals in green. *Rural districts.

Figure S2. Maps for empirical Bayesian estimated incidence rates of URI and LRI at ward level

a) Empirical Bayesian estimated incidence rates (EBR) per 1000 population of upper respiratory infections (URI) with locations of hospitals in green, b) EBR of lower respiratory infections (LRI) per 1000 population with locations of hospitals in green. CH1: Children Hospital 1, CH2: Children Hospital 2; HTD: Hospital for Tropical Diseases. District names and district boundaries (grey) are shown. *Rural districts.

Figure S3. Diagnostic plots of the full generalized additive mixed models (GAMM) for URI and LRI

Left panel: upper respiratory infection (URI); right panel: lower respiratory infection (LRI).

Figure S4. Autocorrelation function (ACF) and partial autocorrelation function (PACF) of the residuals of the full generalized additive mixed models (GAMM) for URI and LRI

Left panel: upper respiratory infection (URI); right panel: lower respiratory infection (LRI).

For peer review only

For peer review only - http://bmjopen.bmj.com/site/about/guidelines.xhtml

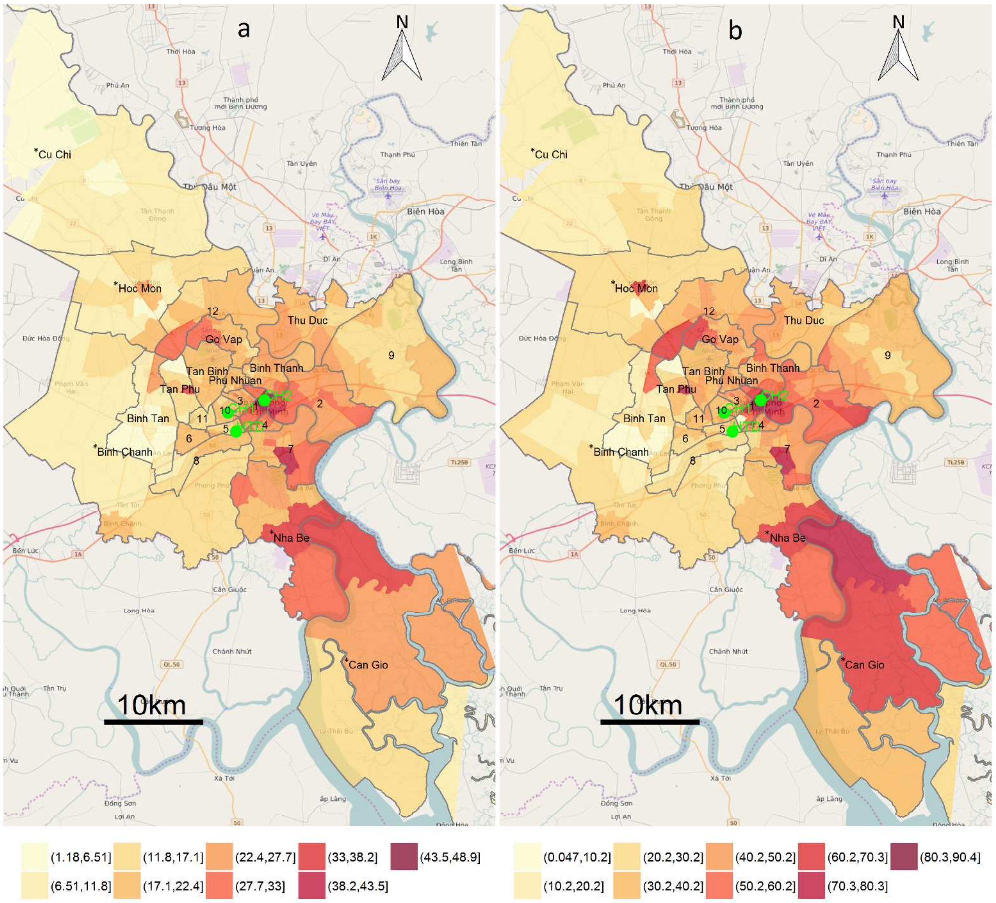

Figure 1

only

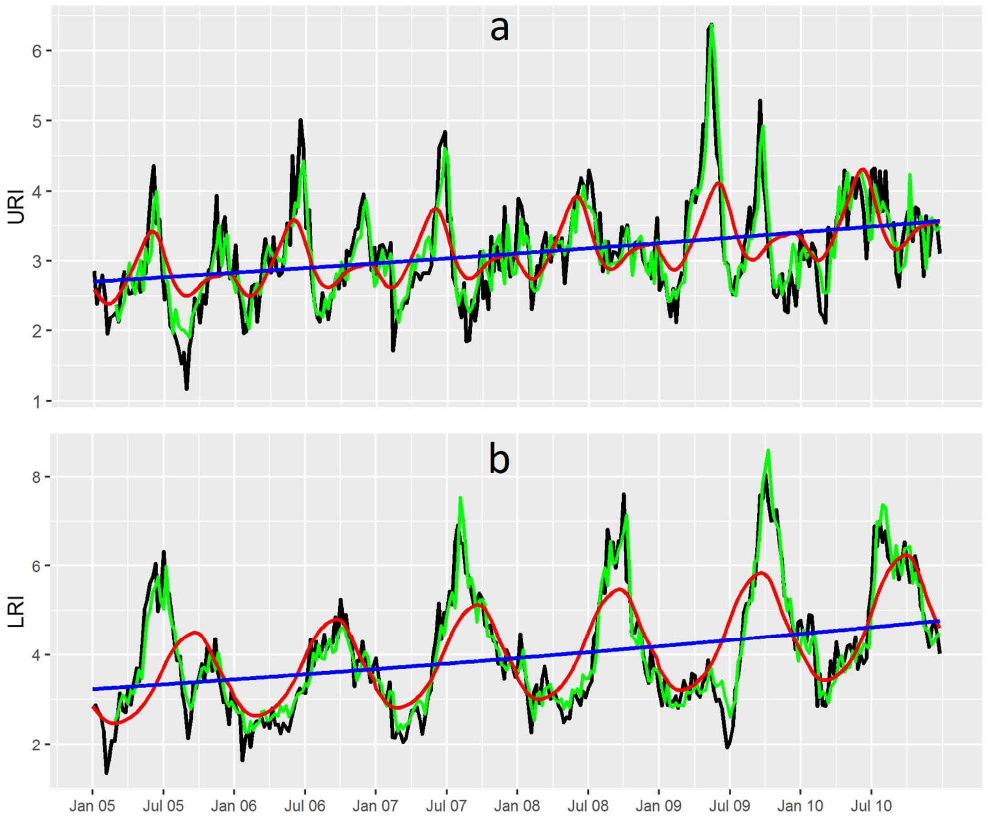

Figure 2

w only

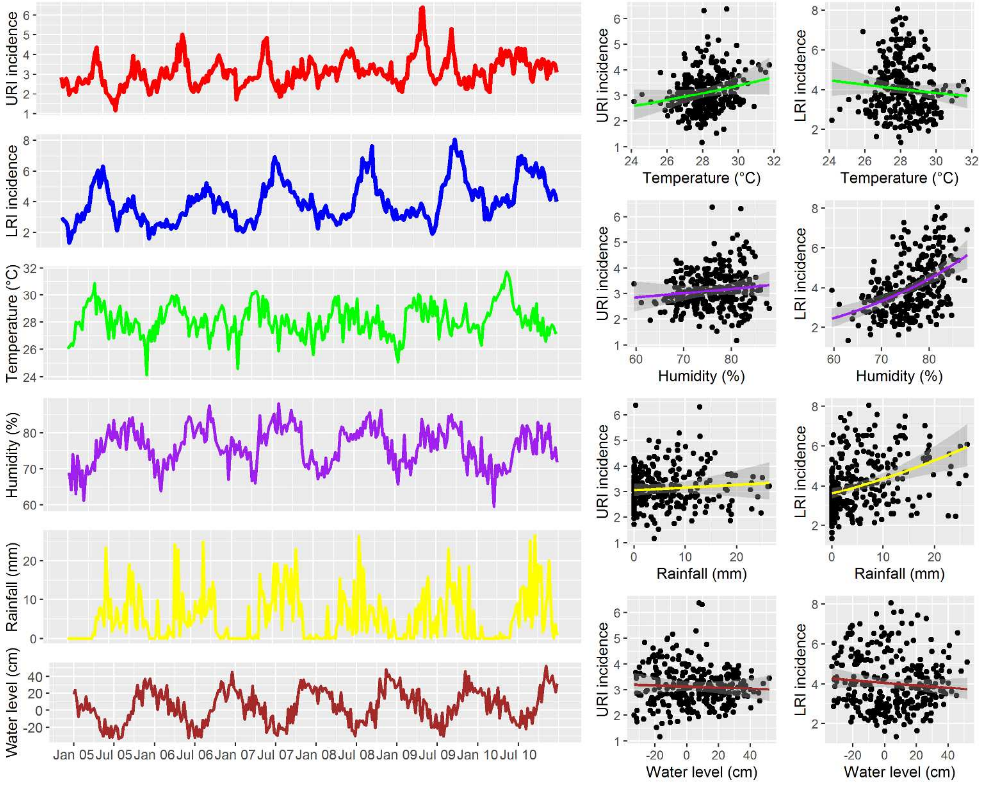
URI incidence
LRI incidence
Temperature (°C)
Humidity (%)
Rainfall (mm)
Water level (cm)
Jan 05 Jul 05 Jan 06 Jul 06 Jan 07 Jul 07 Jan 08 Jul 08 Jan 09 Jul 09 Jan 10 Jul 10
Temperature (°C)
Humidity (%)
Rainfall (mm)
Water level (cm)

Figure 3

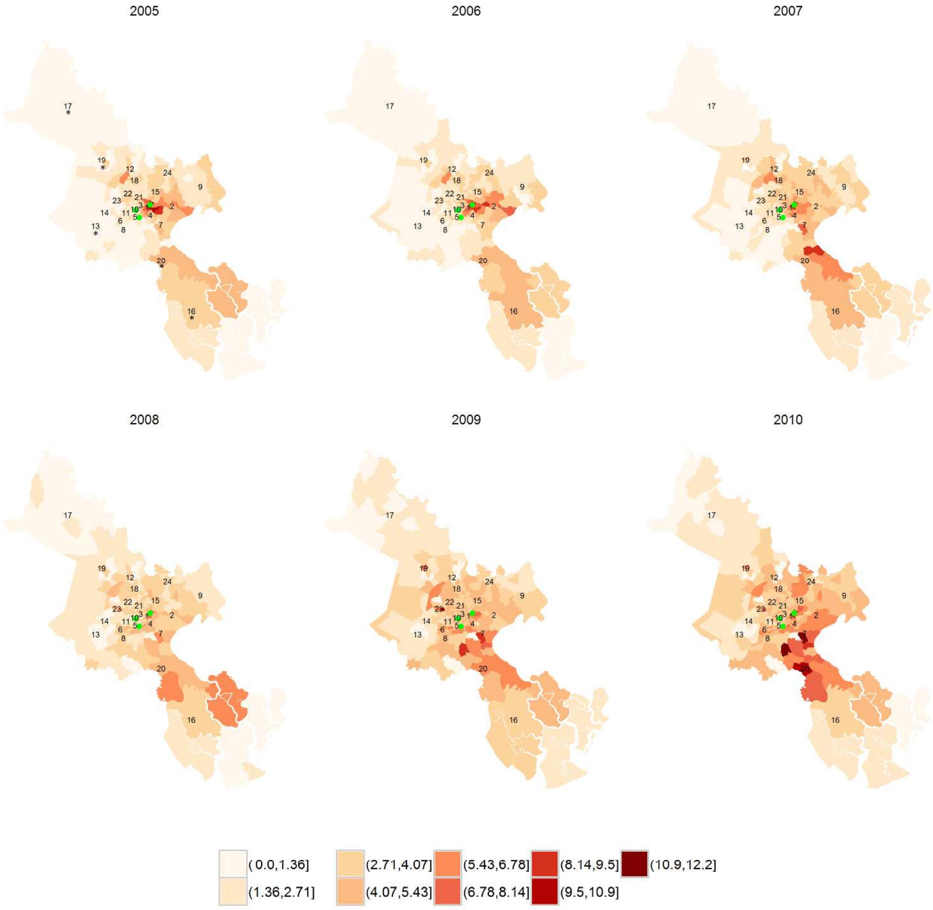

Figure S1

only

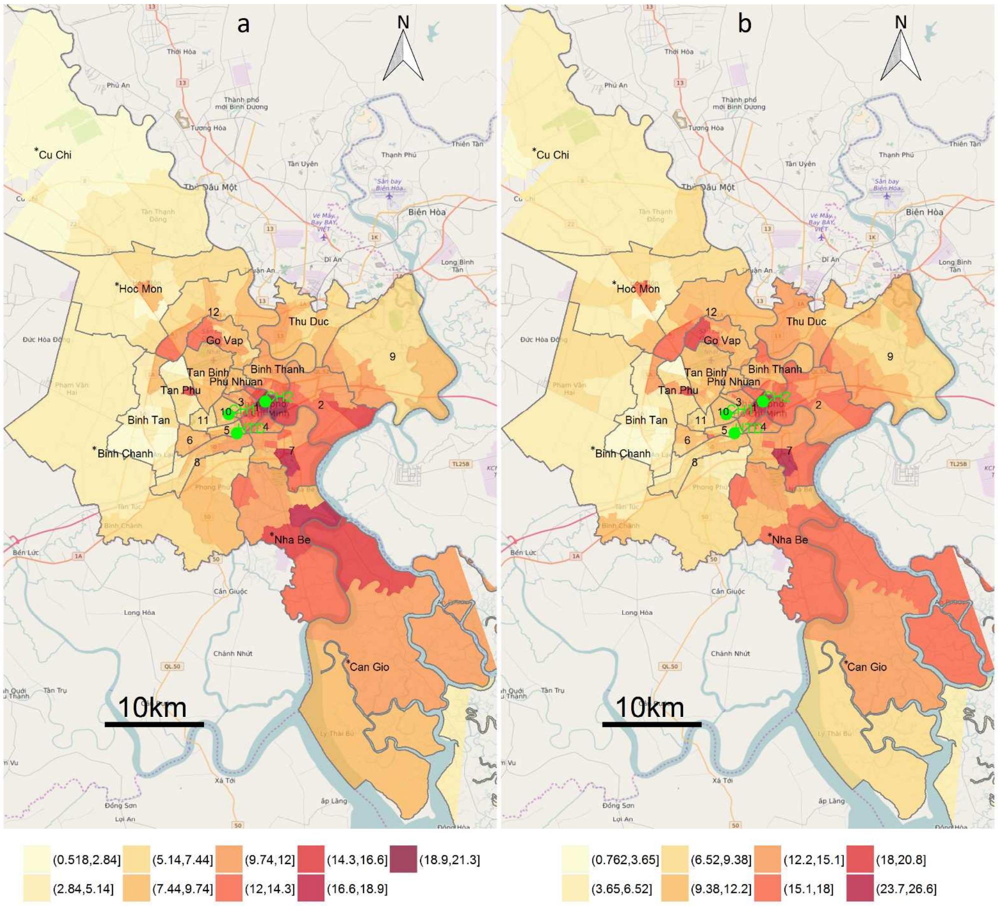

Figure S2

only

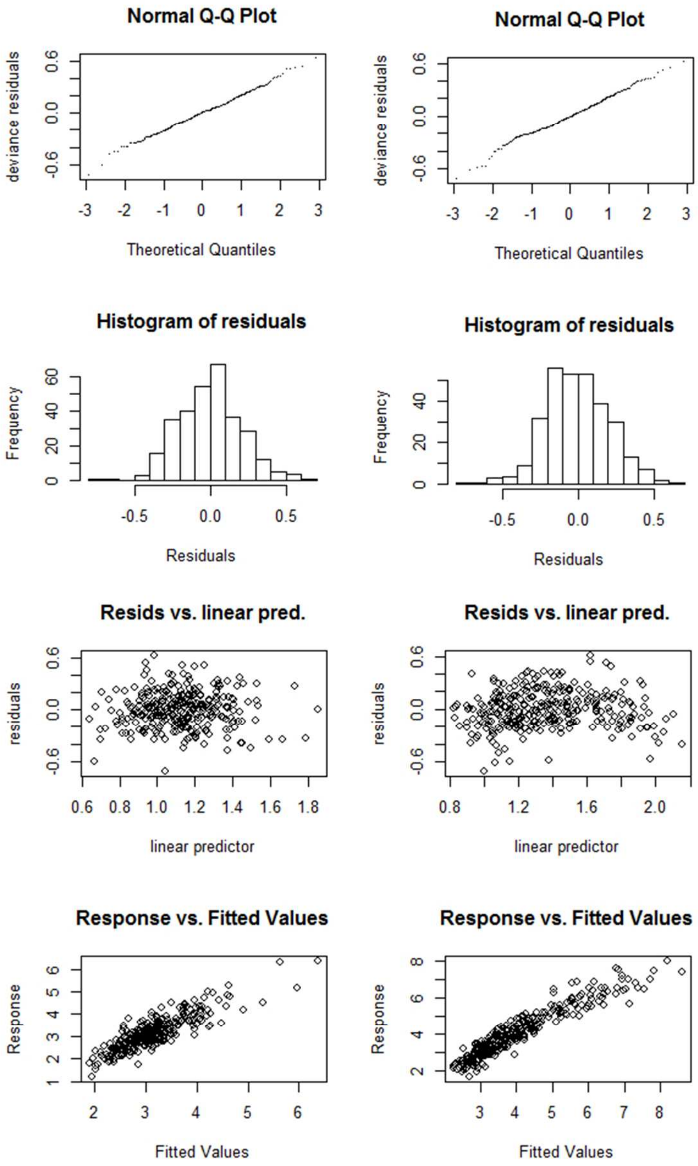
Normal Q-Q Plot
deviance residuals
Theoretical Quantiles
Normal Q-Q Plot
deviance residuals
Theoretical Quantiles
Histogram of residuals
Frequency
Residuals
Histogram of residuals
Frequency
Residuals
Resids vs. linear pred.
residuals
linear predictor
Resids vs. linear pred.
residuals
linear predictor
Response vs. Fitted Values
Response
Fitted Values
Response vs. Fitted Values
Response
Fitted Values

Figure S3

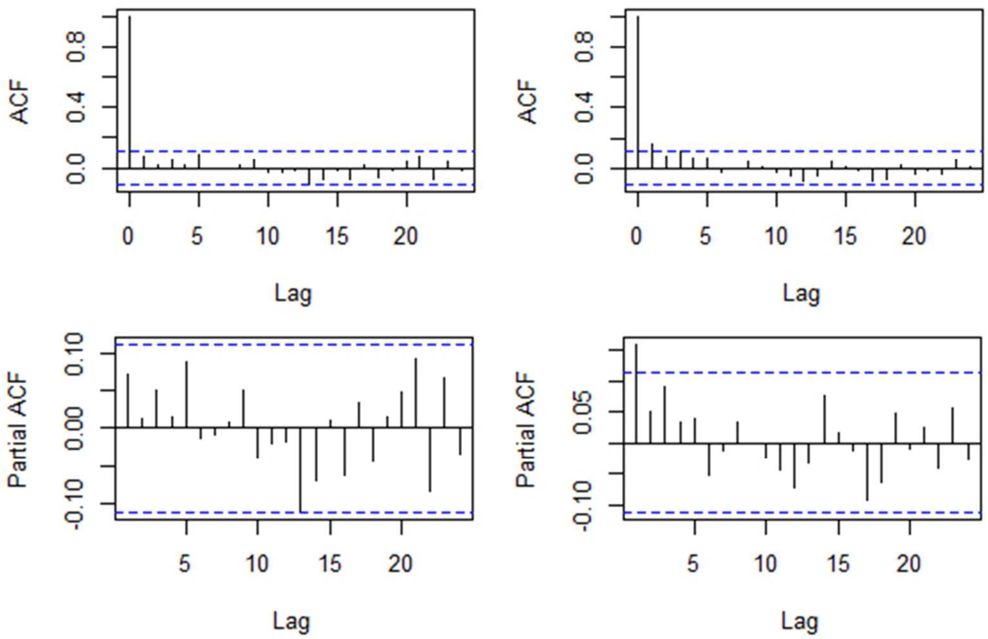

Figure S4

For peer review only

BMJ Open

A retrospective analysis assessing the spatial and temporal distribution of pediatric acute respiratory tract infections in Ho Chi Minh City, Vietnam

Journal:	*BMJ Open*
Manuscript ID	bmjopen-2017-016349.R1
Article Type:	Research
Date Submitted by the Author:	06-Nov-2017
Complete List of Authors:	Ho, Nhan Thi; Oxford University Clinical Research Unit, Enteric infections<br>Thompson, Corinne; Oxford University Clinical Research Unit, Enteric infections<br>Nhan, Le Nguyen Thanh; Children's hospital one<br>Van, Hoang Minh Tu; Children's hospital one<br>Dung, Nguyen Thanh; The Hospital for Tropical Diseases<br>Tran My, Phuc; Oxford University Clinical Research Unit, Enteric infections<br>Quang, Vo Minh; The Hospital for Tropical Diseases<br>Minh, Ngo Ngoc Quang; The Hospital for Tropical Diseases<br>Tuan, Tran Anh; Oxford University Clinical Research Unit, Enteric infections<br>Hung, Nguyen Thanh; Children's hospital one<br>Tuan, Ha Manh ; Children's hospital 2<br>Vinh Chau, Nguyen Van; The Hospital for Tropical Diseases<br>Wolbers, Marcel; Oxford University Clinical Research Unit, Enteric infections<br>Thwaites, Guy; Oxford University Clinical Research Unit, Enteric infections<br>Choisy, Marc; Oxford University Clinical Research Unit, Enteric infections<br>Baker, Stephen; Oxford University Clinical Research Unit, Enteric infections
**Primary Subject Heading**:	Respiratory medicine
Secondary Subject Heading:	Epidemiology, Infectious diseases, Paediatrics
Keywords:	Acute respiratory infections, upper respiratory infections, lower respiratory infections, spatial distribution, temporal patterns, pediatrics

SCHOLARONE™
Manuscripts

A retrospective analysis assessing the spatial and temporal distribution of pediatric acute respiratory tract infections in Ho Chi Minh City, Vietnam

Nhan Thi Ho [1†#], Corinne Thompson [1,2,3†], Le Nguyen Thanh Nhan [4], Hoang Minh Tu Van [5], Nguyen Thanh Dung [6], Tran My Phuc [1], Vo Minh Quang [6], Ngo Ngoc Quang Minh [4], Tran Anh Tuan [4], Nguyen Thanh Hung [4], Ha Manh Tuan [5], Nguyen Van Vinh Chau [6], Marcel Wolbers [1,2], Guy E Thwaites [1,2], Marc Choisy [7] and Stephen Baker [1,2,8*]

[1] Oxford University Clinical Research Unit, Wellcome Trust Major Overseas Program, Ho Chi Minh City, Vietnam

[2] Centre for Tropical Medicine and Global Health, Nuffield Department of Medicine, University of Oxford, United Kingdom

[3] The London School of Hygiene & Tropical Medicine, United Kingdom

[4] Children's Hospital 1, Ho Chi Minh City, Vietnam

[5] Children's Hospital 2, Ho Chi Minh City, Vietnam

[6] Hospital for Tropical Diseases, Ho Chi Minh City, Vietnam

[7] Institute of Research and Development, Vietnam

[8] The department of Medicine, the University of Cambridge, Cambridge, United Kingdom

† These authors contributed equally to this work.

Current institution: Columbia University Medical Center, New York, United States

* Corresponding Author: Prof. Stephen Baker, Oxford University Clinical Research Unit, 764 Vo Van Kiet, Quan 5, Ho Chi Minh City, Vietnam. Tel: +84 89241761 Fax: +84 89238904 sbaker@oucru.org

Author email addresses: Nhan Thi Ho: nth2111@cumc.columbia.edu, Corinne Thompson: corinne.thompson1@gmail.com, Le Nguyen Thanh Nhan: nhanlnt@oucru.org, Hoang Minh Tu Van: vanhmt@oucru.org, Nguyen Thanh Dung: bsdungbvbnd@gmail.com, Tran My Phuc:

For peer review only - http://bmjopen.bmj.com/site/about/guidelines.xhtml

phuctm@oucru.org, Vo Minh Quang: bsvominhquang@gmail.com, Ngo Ngoc Quang Minh: minhnnq@yahoo.com, Tran Anh Tuan: drtat@hotmail.com, Nguyen Thanh Hung: drthanhhung@gmail.com, Ha Manh Tuan: manhtuanzu@yahoo.com, Nguyen Van Vinh Chau: chaunvv@oucru.org, Guy Thwaites: gthwaites@oucru.org, Marc Choisy: mchoisy@oucru.org, Marcel Wolbers: marcel.wolbers@gmx.ch, Stephen Baker: sbaker@oucru.org

For peer review only

For peer review only - http://bmjopen.bmj.com/site/about/guidelines.xhtml

Abstract

Background: Acute respiratory infections (ARIs) are the leading cause of morbidity and mortality in young children in developing countries. Using routine hospital data we aimed to examine the spatial distribution, temporal trends, and climatic risk factors of pediatric ARIs in Vietnam.

Methods: Data from hospitalized pediatric (<16 years old) patients with ARIs residing in Ho Chi Minh City (HCMC) between 2005-2010 were retrieved from the two main Children's Hospitals and the Hospital for Tropical Diseases in HCMC. Spatial mapping and time series analysis were performed after disaggregating the data into upper respiratory infections (URIs) and lower respiratory infections (LRIs).

Results: Over the study period, there were 155,999 pediatric patients admitted with ARIs (33% of all admissions). There were 68,120 URIs (14%) and 87,879 LRIs (19%). The most common diagnoses were acute pharyngitis (28% of all ARI), pneumonia (21%), bronchitis (18%), and bronchiolitis (16%). A significant increasing incidence trend was found for URIs (incidence rate ratio for 1 week increase in time (RR)=1.09, 95%CI=1.02-1.17) and LRIs (RR=1.08, 95%CI=1.006-1.16). The weekly URI incidence peaked in May-June and was significantly associated with lags in weekly URI incidence and the average humidity, rainfall, and water level. The weekly LRI incidence exhibited significant seasonality ($p<0.0001$), with an annual peak in September-October and was significantly associated with lags in weekly LRI incidence and lags in weekly average temperature, rainfall, and water level.

Conclusions: ARIs were the leading cause of childhood hospitalization in HCMC, Vietnam. The incidence of ARIs was higher in the wet season and higher in specific HCMC districts. These results may guide health authorities in where and when to effectively allocate resources for the prevention and control of ARIs.

For peer review only - http://bmjopen.bmj.com/site/about/guidelines.xhtml

For peer review only

Strengths and limitations of the study

- Using routine data from electronic hospital databases we were able to retrieve (based on International Classification of Disease version 10 (ICD10) codes) access information on pediatric admissions due to acute respiratory infections (ARIs) over 6 year period in Ho Chi Minh City to examine the spatial distribution, temporal patterns, and climatic risk factors of pediatric ARIs.
- Pediatric ARI admissions were mapped and compared with all other pediatric admissions to assess the spatial distribution of ARI admissions in comparison with the spatial referral pattern of the study hospitals.
- Data were stratified in two clinically differentiable groups: upper respiratory infections (URIs) and lower respiratory infections (LRIs) to utilize the general ICD10 codes in the databases.
- Our findings were generated using hospitalized patient data and thus might not be generalizable for all outpatients. This may be particularly true for URIs, as many patients with URIs do not require hospitalization.
- There was limited data on the specific pathogens associated with ARIs in the databases. URIs and LRIs are comprised of various diseases associated with a wide range of pathogens, which may have different spatial temporal patterns.

Key words

Acute respiratory infections, upper respiratory infections, lower respiratory infections, spatial distribution, temporal patterns, pediatrics

For peer review only

Introduction

Acute respiratory tract infections (ARIs) are comprised of upper respiratory tract infections (URIs) and lower respiratory tract infections (LRIs). URIs are associated with infections at or above the larynx, are most commonly induced by viruses and include rhinitis (the common cold), sinusitis, ear infections, acute pharyngitis, epiglottitis, and laryngitis. LRIs are comprised of bacterial or viral infections occurring below the larynx and encompass bronchitis, bronchiolitis, and pneumonia [1]. ARIs are the leading cause of morbidity and mortality in young children [2–4], accounting for >18% of all childhood deaths globally [5]. In 2010, there were approximately 120 million episodes and 1.3 million deaths associated with pneumonia worldwide, the vast majority of these occurred in Southeast Asia and sub-Saharan Africa [5,6]. In industrializing regions, ARIs in children aged <5 years are generally more severe than in older children and adults, resulting in a higher case-fatality than in industrialized countries [1,7]. This disparity in mortality rate is multifactorial and associated with differing etiological agents, available therapies, exposure frequencies, and host immunity.

In Ho Chi Minh City (HCMC), Vietnam, ARIs are amongst the most common cause of childhood hospitalizations [8–10]. The common identified pathogens of hospitalized ARI in HCMC include respiratory syncytial virus (RSV), rhinovirus, coronavirus, influenza, and bocavirus [8–10], in addition to the bacterial pathogens *Streptococcus pneumoniae* and *Haemophilus influenzae* type b (Hib) [11,12]. With limited access to costly vaccines for bacterial etiologies and a lack of vaccines for viral causes of ARIs, a greater understanding of the risk factors and sub-populations at risk of ARIs in HCMC is necessary to effectively focus existing prevention measures such as the encouragement of breastfeeding, hand washing with soap, and a reduction in household air pollution [2].

There are data to suggest that the incidence of ARIs is associated with specific spatial, meteorological, and socio-demographic risk factors [13–16]. However, specific temporal and climatic risk factors for both URIs and LRIs in HCMC have not been investigated. Here we aimed to examine the spatial

For peer review only - http://bmjopen.bmj.com/site/about/guidelines.xhtml

distribution, temporal patterns, and climatic risk factors for hospitalized pediatric ARIs in HCMC. This analysis was performed to generate a better understanding of the factors that may influence ARIs and in order to guide healthcare resources for this common pediatric disease syndrome.

Methods

Data sources

Data for this study was derived from the electronic hospital databases of all pediatric (<16 years) inpatient admissions to three large tertiary hospitals located in central HCMC: Children's Hospital 1 (CH1), Children's Hospital 2 (CH2), and the Hospital for Tropical Diseases (HTD). Data from the two children hospitals were collected between 2005 and 2010 (inclusive) while the data from HTD were available from 2008 to 2010 only.

ARIs are caused by a range of differing pathogens and may have different spatiotemporal patterns and associations with climate and other covariates. Therefore, we allocated respiratory infections into two major clinically differentiable groups based on the International Classification of Disease version 10 (ICD10) codes version 2016 outlined by the World Health Organization (WHO): 1) URIs were amalgamated under the ICD10 codes J00:J06 and J9:J11 for influenza like illnesses and other acute upper respiratory infections and 2) LRIs, which incorporated the ICD10 codes J12:J18 and J20:J22 for pneumonia and other acute lower respiratory infections. For the purposes of this study the data from patients with the selected ICD10 codes who resided in HCMC were extracted and analyzed. Each patient record included data regarding age, sex, date of admission and discharge, ICD10 code at discharge, and residential address.

HCMC is a large economic center in the South of Vietnam with a population of >8 million people. Geographically, it is divided into 24 districts (land areas ranging from 4.18 km^2- 704.22 km^2), which are subdivided in 322 smaller administrative wards. There are 5 rural districts (population density ranging

For peer review only - http://bmjopen.bmj.com/site/about/guidelines.xhtml

from 100 persons/km^2 to 3,326 persons/km^2) and 19 urban districts (population density ranging from 2,360 persons/km^2 to 45,582 persons/km^2). There are at least one local (district) hospital in each district, which serves both outpatients and inpatients of all ages. However, most local hospitals, especially those in urban districts receive mostly adult outpatients. There are two central hospitals (CH1 and CH2) that specialize in serving the pediatric population in HCMC. Another further central hospital (HTD) specializes in infectious diseases and also receives pediatric patients. While pediatric outpatients in HCMC may choose to visit local or private healthcare facilities or tertiary hospitals, most pediatric patients requiring hospitalization in HCMC are referred or self-referred to one of the three study hospitals. Therefore, the three study hospitals (CH1, CH2, and HTD) receive the majority of pediatric inpatients from HCMC.

There are two main seasons in HCMC: a dry season from December to April and a rainy season from May to November. Population and district characteristic data (urban vs. rural districts, district population density, district economic indicators (expenditure of district budget, revenues of district budget), district agricultural indicators (number of pigs in herds > 2 months of age by district, % area of district used as rice paddy), and district education indicator (number of primary and secondary schools per district) were obtained from the HCMC Statistical Office [17]. As population figures for those aged <16 years per ward or district was not obtainable, the total population was used to estimate disease incidence. Patient addresses in HCMC were geocoded to districts and wards. City level weekly average climate data during 2005–2010 were obtained from the Ministry of Natural Resources and Environment of Vietnam including relative humidity (median=76.5%, range=61.7% to 88%), temperature (median=28.1°C, range=24.4°C to 31.8°C), rainfall (median=24.4mm, range=0mm to 230.7mm) and water level of the Dong Dien River (median=4.93cm, range= -34.43cm to 49.43cm) [18].

For peer review only - http://bmjopen.bmj.com/site/about/guidelines.xhtml

182 *Spatial mapping*

183 The variance of raw ward ARI incidence rates (number of ARI cases per 1,000 ward total population for
184 the whole study period) may largely vary due to the large variation in ward population size in HCMC. To
185 take into account the spatial heterogeneity of variance and any dependence of ARI incidence rates
186 between wards, ward ARI incidence rates were smoothed using local empirical Bayes estimates for rates
187 reduced to a neighborhood mean [19]. The neighborhoods were selected using the neighborhood list
188 based on wards with contiguous boundaries (R package spdep version 0.5-88) [20]. Kulldorff and
189 Nagarwalla's method over the centroids was be used to scan for clusters of ARIs [21]. This method allows
190 the detection of possible cluster of any size, at any location in a population with inhomogeneous spatial
191 density of HCMC. A Poisson generalized linear model was used to explore the relationship between
192 district ARI incidence and district characteristics. Confidence intervals and tests were based on robust
193 standard errors to control for mild violation of the distributional assumption that the variance equals the
194 mean [22].

195

196 *Time series analysis*

197 Citywide ARI weekly incidences (number of cases per 1,000 population) were examined for seasonality,
198 time trends and associations with climatic covariates by Poisson generalized additive mixed models
199 (GAMM) implemented in the R package mgcv version 1.8-7 [23,24]. The seasonal cycles and time trends
200 of weekly ARI incidence were evaluated by the basic GAMM models containing the week of year (Ws=1
201 to 52)) as cyclic cubic regression splines and the week of the whole study period (Wt=1 to 312)) as cubic
202 regression spline. As weekly ARI incidence may be associated with the ARI incidence and climatic
203 condition of the previous weeks, in addition to the elements in the basic model, the full GAMM model
204 contained lags of ARI weekly incidence up to eight weeks, concurrent climatic condition and lags of
205 climatic condition up to 8 weeks. The performance of the GAMM models was evaluated using adjusted
206 R-squared and model diagnostic plots. Temporal correlation of the model residuals was evaluated by
207 autocorrelation function (ACF) and partial autocorrelation function (PACF).

For peer review only - http://bmjopen.bmj.com/site/about/guidelines.xhtml

All analyses and results were based on the records with available relevant information (complete cases). Spatial mapping and statistical analyses were stratified by URIs and LRIs and all analyses were performed in R version 3.2.2 [25].

Results

Basic features of acute respiratory infections in the pediatric population of Ho Chi Minh City

From 2005 to 2010, there were 155,999 children (<16 years old) residing in HCMC admitted to the three study hospitals for ARIs (33% of all admissions); 323,245 patients were admitted for conditions other than ARIs. There were 68,120 patients with ICD10 codes synonymous with acute URIs (14% of all admissions) and 87,879 patients with ICD10 codes associated with acute LRIs (19% of all admissions). The proportion of males (62%) and females (38%) was comparable between URIs and LRIs. The median age of the patients was 1.5 years (interquartile range [IQR]: 0.7-2.9 years). However, patients with LRIs were significantly younger than those with URIs (median age in years (IQR) = 1.1 (0.5, 2.1) vs. 2.1 (1.1, 3.9)), Wilcoxon's test $p<0.0001$) and LRI patients were hospitalized significantly longer than those with URIs (median days of hospitalization (IQR) = 6.0 (4.0, 9.0) vs. 4.0 (2.0, 5.0), Wilcoxon's test $p<0.0001$) (Table 1).

Overall, the most common diagnoses were acute pharyngitis (28% of all ARIs), pneumonia (21% of all ARIs), bronchitis (18% of all ARIs) and bronchiolitis (16% of all ARIs). Among the URI patients, the majority of patients were diagnosed with acute pharyngitis (63% of all URIs), acute upper respiratory infections of multiple and unspecified sites (14%), acute tonsillitis (8%), acute laryngitis and tracheitis (6%), and acute nasopharyngitis (6%). Among the LRI patients, the most common diagnoses were pneumonia with an unspecified organism (37% of all LRIs), acute bronchitis (32%), acute bronchiolitis (29%), and bacterial pneumonia not elsewhere classified (2%) (Table 1).

For peer review only - http://bmjopen.bmj.com/site/about/guidelines.xhtml

The spatial distribution of pediatric acute respiratory infections in Ho Chi Minh City

The raw ward-level incidence rates of ARIs for the entire study period ranged from zero to 50 per 1,000 total population (median (IQR) = 18.8 (14.2, 23.5)) and the smoothed ward level empirical Bayesian estimated ARI incidence rates ranged from 1.2 to 48.8 ARI cases per 1,000 population (median (IQR) = 18.6 (14.7, 23.7)). High incidence rates (>30 cases/1,000 population) of ARI were observed not only in the wards surrounding the three study hospitals (wards in districts 1 and 4 in central HCMC) but also in multiple wards located outside the central region of the city, specifically wards located in districts 2, 7, and Nha Be (observed vs. expected incidence rate ratio >1.4, $p<0.0001$ using Kulldorff and Nagarwalla's scan) (Figure 1a). Notably, the spatial distribution of ARIs and diseases other than ARI were comparable (Figure 1a and 1b). We observed an increase with time in ward incidence of ARIs in the majority of the wards with the most apparent high incidences in wards in the south of the city (Figure S1). We additionally found the spatial distribution of URIs and LRIs were comparable (Figure S2).

The district level incidence over the entire study period of ARIs was significantly lower in the districts located further away from the hospitals (RR for 2 times increase in distance to hospital = 0.88; 95% CI = 0.80, 0.96) and in rural districts (RR for urban vs. rural districts= 1.54; 95% CI = 1.10, 2.15) (Figure 1a). Other district characteristics were not significantly associated with district ARI incidence.

Temporal patterns and climatic risk factors of pediatric acute respiratory infections

The mean of weekly incidence of URIs for the study period was 3.12/1,000 population. Weekly URI incidence showed a relatively small increase with time, with a difference in mean weekly URI incidence at the end and the start of the study period of 0.86 patients per 1,000 population. The basic Poisson GAMM model indicated that URI admissions in the study hospitals exhibited a significant increasing trend with time ($p<0.0013$) and seasonality ($p<0.0001$) with a peak in May to June and a smaller peak in December to January (Figure 2a).

For peer review only - http://bmjopen.bmj.com/site/about/guidelines.xhtml

The mean weekly LRI incidence for the study period was 4.02/1,000 population. There was a significant increase of weekly LRI incidence over time (p=0.0037) with a substantial difference in the mean weekly LRI incidence at the end vs. the start of the study period of 1.54 patients/1,000 population. The seasonal cycle of LRIs (p<0.0001) was more obvious than that of URIs, with a single, wide annual peak in the months of September and October (Figure 2b).

In a univariate analysis with Poisson generalized linear model, we found that weekly URI incidence was significantly associated with weekly average temperature (RR for 1 degree increase of temperature=1.048, 95%CI=1.030-1.066) and humidity (RR for 1% increase in relative humidity=1.006, 95%CI= 1.001-1.011). Further, weekly LRI incidence was significantly associated with weekly average humidity (RR=1.030, 95%CI=1.024-1.036) and rainfall (RR for 1mm increase in rainfall= 1.019, 95%CI=1.014-1.024) (Figure 3). However, after controlling for seasonality, time trend, lags of weekly incidence, and other climatic covariates in the full GAMM model, the weekly URI incidence was significantly associated with one and two week lags in URI incidence (RR=1.202, 95%CI=1.159-1.245 and RR=1.063, 95%CI=1.019-1.109, respectively), one and three week lags in weekly average humidity (RR=1.009, 95%CI=1.003-1.014 and RR=0.990, 95%CI=0.984-0.996, respectively), a three week lag in weekly average rainfall (RR=1.006, 95%CI=1.003-1.009) and a four week lag in weekly average river water level (RR for 1 cm increase in water level=1.003, 95%CI=1.002-1.005) (Table 2).

After adjusting for other factors in the full GAMM model, weekly LRI incidence was significantly associated with a one and two week lags in LRI incidence (RR=1.173, 95%CI=1.138-1.208 and RR=1.053, 95%CI=1.015-1.092 respectively), a one week lag in weekly average temperature (RR=0.976, 95%CI=0.953-0.998), a one week lag in weekly average rainfall (RR=1.004, 95%CI=1.001-1.007), and a one week lag in weekly average water level (RR=0.998, 95%CI=0.997-1.000) (Table 2). The time trends remained significant for both URIs (p=0.0096) and LRIs (p=0.0345) while only LRIs exhibited

For peer review only

For peer review only - http://bmjopen.bmj.com/site/about/guidelines.xhtml

significant seasonality (p<0.0001) in the full GAMM models. The full GAMM models fitted the data well for both URIs (adjusted R squared =0.72) and LRIs (adjusted R squared =0.88) (Figure 2 and Figure S3). Temporal correlation of weekly incidence was well controlled for URIs and also relatively well controlled for LRIs in the full GAMM models (Figure S4).

Discussion

Our study examined the spatial distribution, temporal patterns, and climatic risk factors of ARIs in HCMC, Vietnam. The findings were based on the data of a large number of ARI inpatients retrieved from routinely collected electronic hospital databases over a 6-year period. Our data showed that ARIs accounted for one third of all hospital admissions over the study period and were the most common cause of hospitalized diseases in children in HCMC. The spatial distribution of ARIs was comparable to the spatial referral patterns of study hospitals in HCMC (the spatial distribution of diseases other than ARIs). High incidence of ARIs was found not only in districts close to the study hospitals (districts 1 and 4) but also districts 2, 7, and Nha Be, which are located further away from three study hospitals. This finding is likely a result of urbanization or better economic status of those living in these districts.

The observed increase in hospitalized ARI incidence with time may be explained by better healthcare awareness within the population, through better access to tertiary hospitals, better healthcare policies (free healthcare for children aged <6 years was initiated in Vietnam), or a real increase in ARI incidence. The citywide increase of ARI incidence, especially LRIs, may also be attributed to an increase in air pollution. Pollution was shown to be associated with an increase in LRI incidence in study conducted by the Collaborative Working Group on Air Pollution, Poverty, and Health in HCMC [26]. The number of beds in these tertiary hospitals did not increase over the study period, and the policy of these hospitals is to accommodate all patients visiting the hospitals regardless of the capacity of the hospital and staff. An increase in ARI admission indicates an increase in burden for these tertiary hospitals and an increased workload for healthcare, which may impact negatively on the quality of care. Therefore, there should be

For peer review only - http://bmjopen.bmj.com/site/about/guidelines.xhtml

effective policies to improve the allocation of healthcare resources in these hospitals as well as to improve the quality and capacity of local healthcare facilities to share this increasing disease burden.

The weekly incidence of both URIs and LRIs showed significant association with 1 and 2 week lags of incidence indicating that temporal correlation might play an important role in URI and LRI incidence. Furthermore, we additionally noted a difference in seasonal patterns of URIs (two small peaks a year) and LRIs (one strong peak a year) and a difference in association patterns with climatic factors of URIs and LRIs. These findings suggested that climatic factors might differentially influence the pathogens causing URIs and LRIs in HCMC. The association of URI incidence with one and three week lags of weekly average humidity was consistent with the association between the seasonality of absolute humidity and influenza-like illness (ILI) in Vietnam as previously reported [27]. In addition, the national surveillance of influenza and ILI in Vietnam during the study period exhibited a similar seasonal pattern with two peaks a year at approximately the same months with the two peaks of URIs in our data [28]. The exceptional high peak of URIs without a concurrent peak of LRIs in 2009 also occurred simultaneously with 2009 H1N1. Therefore, influenza and ILI were likely key contributors to the seasonality and the peaks of URIs in our data. Moreover, a study conducted at CH2 investigating viral ARI pathogens found that the peak of RSV infections occurred in rainy season (May-November in HCMC). This period included the peak of LRI months and the larger URI peak [10]. Furthermore, RSV is also thought to predispose infected hosts to bacterial infections [29]. In addition, a sharp increase of LRI incidence was observed soon after the peak of URIs (May-June) suggests that URIs and LRIs share some common epidemiological features. As a consequence, appropriate treatment and transmission control for URIs may impact on the incidence of later hospitalization due to LRIs. As the peaks of hospitalization for LRIs are in September-October, resources for pediatric healthcare should be prepared and local healthcare facilities should be enhanced to share the increase in hospitalization caused by LRIs at tertiary hospitals during this period.

For peer review only - http://bmjopen.bmj.com/site/about/guidelines.xhtml

A large proportion of ARI admissions in these data were URIs, two third of these were diagnosed with acute pharyngitis. These conditions can usually be treated in outpatient settings and do not typically require hospitalization. However, the decision to hospitalize ARI patients in these tertiary hospitals is not strictly based on the WHO Integrated Management of Childhood Illness (IMCI) guidelines and is also dependent on the preferences of caregivers and the subjective patient evaluation of the attending physicians. Therefore, there should stricter policies for applying IMCI guidelines and other clinical criteria to reduce unnecessary URI admissions in these hospitals, as well as to improving outpatient care and follow up.

Our study has limitations as the findings were generated using routine data of from hospitalized patients. Therefore, are finding may be generalizable for outpatients, especially for URIs, as many patients with URI may not be admitted to hospital. The findings for LRIs may be more generalizable as LRIs are more severe than URIs and more likely to be hospitalized. Second, the databases contained mostly general ICD10 codes and very limited specific diagnostic information regarding the ARI pathogens. Different pathogens can cause differing URI and LRI disease presentations and different pathogens may be influenced differently by climatic, environmental, population factors as well as host immunity. Therefore, further surveillance and studies investigating different groups of pathogens for ARIs are necessary.

Conclusion

We found that ARIs accounted for one third of total hospital admission and were the leading cause of hospitalization in children in HCMC, Vietnam. The spatial distribution of hospitalized ARIs was similar to the spatial referral patterns of the study hospitals with high incidences in the districts surrounding the locations of the three study hospitals and also in three districts further out the city. The seasonality of ARIs may be explained by the association of ARI incidence with various climatic factors (temperature, humidity and rainfall) and the seasonality of influenza, ILI, and RSV. The increase of LRI incidence soon after the peak of URIs suggests that URIs and LRIs share common epidemiological factors or LRIs are

For peer review only

For peer review only - http://bmjopen.bmj.com/site/about/guidelines.xhtml

complications of URIs. Understanding the spatial distribution, temporal patterns, and climatic risk factors of ARIs should aid the health authorities in outbreak preparation and assist in allocating resources for the prevention and control of the diseases, especially in settings where vaccines for viral and bacterial pathogens of ARIs are not routinely available.

Acknowledgements

The authors are grateful to the staffs of Children's Hospital 1, Children's Hospital 2, and The Hospital for Tropical Diseases for providing the raw databases used in this study.

Footnotes

Contributors

This study was conceptualized by NTH (Nhan Thi Ho), CT, MC and SB. Data was obtained by NTH, CT, LNTN, HMTV, NTD, TMP, VMQ, NNQM, TAT, NTH2 (Nguyen Thanh Hung), HMT and NVVC. Data was analyzed by NTH, CT, MW, MC. NTH, CT and SB wrote the manuscript with input from all authors. NTH, CT, LNTN, HMTV, NTD, TMP, VMQ, NNQM, TAT, NTH2, HMT, NVVC, MW, GET, MC, SB read and approved the final version of the manuscript.

Funding

This work was supported by the Wellcome Trust Vizions strategic award (WT1093824). SB is a Sir Henry Dale Fellow, jointly funded by the Wellcome Trust and the Royal Society (100087/Z/12/Z). NTH is currently funded by Mervyn Susser’s fellowship at Columbia University Medical Center, US. The funders had no role in study design, data collection, data analysis, data interpretation, writing of the report, or decision to submit the article for publication.

Competing interests

For peer review only - http://bmjopen.bmj.com/site/about/guidelines.xhtml

For peer review only

We have read and understood BMJ policy on declaration of interests and declare that we have no competing interests.

Ethical approval

Ethical approval was granted from all of the three study hospitals: Children Hospital 1, Children Hospital 2 and the Hospital for Tropical Diseases.

Data sharing statement

Data will be available upon reasonable request to corresponding author.

References

1. Simoes EAF, Cherian T, Chow J, Shahid-Salles SARL, John TJ. Acute Respiratory Infections in Children. Disease Control Priorities in Developing Countries. 2nd ed. Washington DC: World Bank; 2006.

2. WHO/UNICEF Ending Preventable Child Deaths from Pneumonia and Diarrhoea by 2025: The Integrated Global Action Plan for Pneumonia and Diarrhoea (GAPPD). Geneva; 2013.

3. Lozano R, Naghavi M, Foreman K, Lim S, Shibuya K, Aboyans V, et al. Global and regional mortality from 235 causes of death for 20 age groups in 1990 and 2010: A systematic analysis for the Global Burden of Disease Study 2010. Lancet. 2012;380: 2095–2128. doi:10.1016/S0140-6736(12)61728-0

4. Williams BG, Gouws E, Boschi-Pinto C, Bryce J, Dye C. Estimates of world-wide distribution of child deaths from acute respiratory infections. Lancet Infectious Diseases. 2002. pp. 25–32. doi:10.1016/S1473-3099(01)00170-0

5. Liu L, Johnson HL, Cousens S, Perin J, Scott S, Lawn JE, et al. Global, regional, and national causes of child mortality: An updated systematic analysis for 2010 with time trends since 2000. Lancet. 2012;379: 2151–2161. doi:10.1016/S0140-6736(12)60560-1

For peer review only - http://bmjopen.bmj.com/site/about/guidelines.xhtml

6. Fischer Walker CL, Rudan I, Liu L, Nair H, Theodoratou E, Bhutta ZA, et al. Global burden of childhood pneumonia and diarrhoea. Lancet. 2013;381: 1405–1416. doi:10.1016/S0140-6736(13)60222-6

7. Global Burden of Disease Pediatrics Collaboration, Kyu HH, Pinho C, Wagner JA, Brown JC, Bertozzi-Villa A, et al. Global and National Burden of Diseases and Injuries Among Children and Adolescents Between 1990 and 2013: Findings From the Global Burden of Disease 2013 Study. JAMA Pediatr. 2016;98121: 1–21. doi:10.1001/jamapediatrics.2015.4276

8. Anders KL, Nguyen HL, Nguyen NM, Thuy NT Van, Van NTH, Hieu NT, et al. Epidemiology and virology of acute respiratory infections during the first year of life: a birth cohort study in Vietnam. Pediatr Infect Dis J. 2015;34: 361–370.

9. Do AHL, van Doorn HR, Nghiem MN, Bryant JE, Hoang TH thi, Do QH, et al. Viral etiologies of acute respiratory infections among hospitalized vietnamese children in Ho Chi Minh City, 2004-2008. PLoS One. 2011;6. doi:10.1371/journal.pone.0018176

10. Tran DN, Trinh QD, Pham NTK, Vu MP, Ha MT, Nguyen TQN, et al. Clinical and epidemiological characteristics of acute respiratory virus infections in Vietnamese children. Epidemiol Infect. 2015; 1–10. doi:10.1017/S095026881500134X

11. Jauneikaite E, Jefferies JM, Hibberd ML, Clarke SC. Prevalence of Streptococcus pneumoniae serotypes causing invasive and non-invasive disease in South East Asia: A review. Vaccine. 2012;30: 3503–3514. doi:10.1016/j.vaccine.2012.03.066

12. Nyambat B, Dang DA, Nguyen HA, Mai TQ, Rani M, Slack MPE, et al. Rapid assessment of Hib disease burden in Vietnam. BMC Public Health. 2011;11: 260. doi:10.1186/1471-2458-11-260

13. du Prel J-B, Puppe W, Gröndahl B, Knuf M, Weigl JAI, Schaaff F, et al. Are meteorological parameters associated with acute respiratory tract infections? Clin Infect Dis. 2009;49: 861–868. doi:10.1086/605435

14. Chen Z, Ji W, Wang Y, Yan Y, Zhu H, Shao X, et al. Epidemiology and associations with climatic conditions of Mycoplasma pneumoniae and Chlamydophila pneumoniae infections among

For peer review only

For peer review only - http://bmjopen.bmj.com/site/about/guidelines.xhtml

Chinese children hospitalized with acute respiratory infections. Ital J Pediatr. 2013;39: 34. doi:10.1186/1824-7288-39-34

15. Welliver R. The relationship of meteorological conditions to the epidemic activity of respiratory syncytial virus. Paediatr Respir Rev. 2009;10: 6–8. doi:10.1016/S1526-0542(09)70004-1

16. Cardoso MRA, Cousens SN, de Góes Siqueira LF, Alves FM, D'Angelo LA V. Crowding: risk factor or protective factor for lower respiratory disease in young children? BMC Public Health. 2004;4: 19. doi:10.1186/1471-2458-4-19

17. Statistical Office in Ho Chi Minh City. Statistical Yearbook of Ho Chi Minh City 2011. Ho Chi Minh City: Ho Chi Minh City Statistical Office; 2012.

18. Loc PD. Ministry of Natural Resources and Environment of the Socialist Republic of Vietnam. Ha Noi; 2013.

19. Cressie N. Smoothing Regional Maps Using Empirical Bayes Predictors. Geogr Anal. 1992;24: 75–95. doi:10.1111/j.1538-4632.1992.tb00253.x

20. Bivand R, Hauke J, Kossowski T. Computing the jacobian in gaussian spatial autoregressive models: An illustrated comparison of available methods. Geogr Anal. 2013;45: 150–179. doi:10.1111/gean.12008

21. Kulldorff M, Nagarwalla N. Spatial disease clusters: detection and inference. Stat Med. 1995;14: 799–810. doi:10.1002/sim.4780140809

22. Cameron AC, Trivedi PK. Microeconometrics using Stata. Stata Press books. 2009;5: 706. doi:10.1016/S0304-4076(00)00050-6

23. Lin X, Zhang D. Inference in generalized additive mixed models by using smoothing splines. J R Stat Soc Ser B. 1999;61: 381–400.

24. Wood SN. Generalized additive models: an introduction with R. Wiley Interdiscip. 2006;16.

25. R Core Team. R: A Language and Environment for Statistical Computing [Internet]. R Foundation for Statistical Computing, Vienna, Austria. 2016. doi:10.1017/CBO9781107415324.004

26. HEI Collaborative Working Group on Air Pollution and Health in Ho Chi Minh City P, Le TG,

For peer review only

For peer review only - http://bmjopen.bmj.com/site/about/guidelines.xhtml

Ngo L, Mehta S, Do VD, Thach TQ, et al. Effects of short-term exposure to air pollution on hospital admissions of young children for acute lower respiratory infections in Ho Chi Minh City, Vietnam. Res Rep Health Eff Inst. 2012; 5–83.

27. Seasonality of absolute humidity explains seasonality of influenza-like illness in Vietnam. Epidemics. Elsevier; 2015;13: 65–73. doi:10.1016/J.EPIDEM.2015.06.002

28. Nguyen YT, Graitcer SB, Nguyen TH, Tran DN, Pham TD, Le MTQ, et al. National surveillance for influenza and influenza-like illness in Vietnam, 2006−2010. Vaccine. 2013;31: 4368–4374. doi:10.1016/j.vaccine.2013.07.018

29. Beadling C, Slifka MK. How do viral infections predispose patients to bacterial infections? Curr Opin Infect Dis. 2004;17: 185–191. doi:10.1097/00001432-200406000-00003

For peer review only - http://bmjopen.bmj.com/site/about/guidelines.xhtml

Tables

Table 1. The characteristics and diagnoses for all patients with acute respiratory tract infections upon admission

Characteristic	All ARI patients (N=155,999)	URI (N=68,120)	LRI (N=87,879)	p-value
Male sex [a]	96,930/155,997 (62%)	42,251/68,119 (62%)	54,679/87,878 (62%)	0.43*
Age (year) [b]	1.5(0.7,2.9)	2.1(1.1,3.9)	1.1(0.5,2.1)	<0.0001#
Days in hospital [c]	5.0(3.0,7.0)	4.0(2.0,5.0)	6.0(4.0,9.0)	<0.0001#
**ICD10 code at discharge**				
Acute bronchiolitis (J21)	25,123/155,999 (16%)	0/68,120 (0%)	25,123/87,879 (29%)	
Acute bronchitis (J20)	28,202/155,999 (18%)	0/68,120 (0%)	28,202/87,879 (32%)	
Acute laryngitis & tracheitis (J04)	4,323/155,999 (3%)	4,323/68,120 (6%)	0/87,879 (0%)	
Acute nasopharyngitis (J00)	4,049/155,999 (3%)	4,049/68,120 (6%)	0/87,879 (0%)	
Acute obstructive laryngitis & epiglottitis (J05)	9/155,999 (0%)	9/68,120 (0%)	0/87,879 (0%)	
Acute pharyngitis (J02)	43,148/155,999 (28%)	43,148/68,120 (63%)	0/87,879 (0%)	
Acute sinusitis (J01)	592/155,999 (0%)	592/68,120 (1%)	0/87,879 (0%)	
Acute tonsillitis (J03)	5,123/155,999 (3%)	5,123/68,120 (8%)	0/87,879 (0%)	
Acute upper respiratory infections of multiple & unspecified sites (J06)	9,300/155,999 (6%)	9,300/68,120 (14%)	0/87,879 (0%)	
Bacterial pneumonia, not elsewhere classified (J15)	2,016/155,999 (1%)	0/68,120 (0%)	2,016/87,879 (2%)	
Influenza due to other identified virus (J10)	381/155,999 (0%)	381/68,120 (1%)	0/87,879 (0%)	
Influenza, virus not identified (J11)	1,195/155,999 (1%)	1,195/68,120 (2%)	0/87,879 (0%)	
Pneumonia due to *Haemophilus influenza* (J14)	11/155,999 (0%)	0/68,120 (0%)	11/87,879 (0%)	
Pneumonia due to other infectious organisms, not elsewhere classified (J16)	26/155,999 (0%)	0/68,120 (0%)	26/87,879 (0%)	
Pneumonia due to *Streptococcus pneumonia* (J13)	16/155,999 (0%)	0/68,120 (0%)	16/87,879 (0%)	
Pneumonia in diseases classified elsewhere (J17)	1/155,999 (0%)	0/68,120 (0%)	1/87,879 (0%)	
Pneumonia, organism unspecified (J18)	32,371/155,999 (21%)	0/681,20 (0%)	32,371/87,879 (37%)	
Unspecified acute lower respiratory infection (J22)	7/155,999 (0%)	0/68,120 (0%)	7/87,879 (0%)	
Viral pneumonia, not elsewhere classified (J12)	106/155,999 (0%)	0/68,20 (0%)	106/87,879 (0%)	

For peer review only

For peer review only - http://bmjopen.bmj.com/site/about/guidelines.xhtml

a: data from 155997 patients; b: data from 147,420 patients; c: data from 96,362 patients.*Chi-square test comparing URIs vs. LRIs; #From Wilcoxon's rank sum test comparing URIs vs. LRIs.

ICD10: the International Classification of Disease version 10 (ICD10) codes.

For peer review only

For peer review only - http://bmjopen.bmj.com/site/about/guidelines.xhtml

Table 2. Results of the full generalized additive mixed models (GAMM) for upper respiratory tract infections and lower respiratory tract infections

	URIs				LRIs			
	**RR**	**LL**	**UL**	**P value**	**RR**	**LL**	**UL**	**P value**
Intercept	0.340	0.133	0.869	0.0251	1.421	0.381	5.303	0.6013
Lag 1 week incidence	1.202	1.159	1.245	0.0000	1.173	1.138	1.208	0.0000
Lag 2 week incidence	1.063	1.019	1.109	0.0048	1.053	1.015	1.092	0.0060
Lag 3 week incidence	0.999	0.957	1.042	0.9470	0.987	0.951	1.024	0.4861
Lag 4 week incidence	0.974	0.934	1.017	0.2328	0.998	0.962	1.035	0.9180
Temperature (°C)	1.017	0.996	1.040	0.1178	1.006	0.985	1.028	0.5748
Lag 1 week temperature	0.990	0.966	1.015	0.4480	0.976	0.953	0.998	0.0348
Lag 2 week temperature	1.019	0.994	1.045	0.1389	1.007	0.984	1.030	0.5620
Lag 3 week temperature	1.010	0.985	1.035	0.4470	1.019	0.995	1.043	0.1186
Lag 4week temperature	1.012	0.990	1.035	0.2709	1.015	0.993	1.038	0.1722
Humidity (%)	0.998	0.993	1.004	0.5619	0.998	0.993	1.004	0.5506
Lag 1 week humidity	1.009	1.003	1.014	0.0051	0.997	0.991	1.002	0.2716
Lag 2 week humidity	1.002	0.996	1.007	0.6000	1.001	0.995	1.006	0.7603
Lag 3 week humidity	0.990	0.984	0.996	0.0008	1.000	0.995	1.006	0.9237
Lag 4 week humidity	1.005	0.999	1.010	0.1024	1.000	0.994	1.005	0.8610
Rainfall (mm)	1.000	0.997	1.003	0.9670	1.001	0.998	1.004	0.5615
Lag 1 week rainfall	1.001	0.997	1.004	0.7145	1.004	1.001	1.007	0.0073
Lag 2 week rainfall	1.002	0.998	1.005	0.2979	1.000	0.998	1.003	0.7548
Lag 3 week rainfall	1.006	1.003	1.009	0.0005	1.001	0.998	1.004	0.4927
Lag 4 week rainfall	0.997	0.994	1.001	0.1163	0.999	0.997	1.002	0.6949
Water level (cm)	0.999	0.997	1.000	0.1519	0.999	0.997	1.000	0.0751
Lag 1 week water level	1.001	0.999	1.002	0.2120	0.998	0.997	1.000	0.0111
Lag 2 week water level	0.999	0.998	1.001	0.2488	1.000	0.998	1.001	0.7821
Lag 3 week water level	1.000	0.998	1.001	0.7223	1.000	0.999	1.002	0.9576
Lag 4 week water level	1.003	1.002	1.005	0.0000	1.001	1.000	1.003	0.1473
Time trend (week)	1.092	1.022	1.166	0.0096	1.080	1.006	1.159	0.0345

Results of fixed effects (with only the results of lags up to 4 weeks) are shown.

P values for seasonal cycles from the full GAMM of URIs=0.5177 and LRIs<0.0001 respectively.

Adjusted R-squared of the full GAMM for URIs=0.72 and LRIs=0.88 respectively.

RR: rate ratio; LL and UL: lower and upper limit of 95% confidence interval.

For peer review only

492 **Figure legends**

493 **Figure 1.** Maps for empirical Bayesian estimated incidence rates of acute respiratory tract infections and
494 other diseases at ward level

495 a) Ward level empirical Bayesian estimated incidence rates (EBR) per 1,000 population for ARIs with
496 locations of the 3 study hospitals in green, b) ward level EBR for other diseases rather than ARIs.
497 CH1: Children Hospital 1, CH2: Children Hospital 2; HTD: Hospital for Tropical Diseases. District
498 names and district boundaries (grey) are shown. Rural districts are marked with *.

499

500 **Figure 2.** Plots of the citywide weekly incidence time series for upper respiratory tract infections and
501 lower respiratory tract infections from the generalized additive mixed model (GAMM)

502 Upper panels (a): smoothed seasonal cycles (red line), time trend (blue line) from the basic Poisson
503 GAMM model, fitted values (green line) from the full Poisson GAMM model and observed values (black
504 line) of citywide weekly incidence (number of cases per 1000 population) of URIs. Lower panels (b):
505 smoothed seasonal cycles (red line), time trend (blue line) from the basic Poisson GAMM model and
506 fitted values (green line) from the full Poisson GAMM model and observed values (black line) of
507 citywide weekly incidence of LRIs.

508

509 **Figure 3.** Time series of city wide weekly incidence of upper respiratory tract infections and lower
510 respiratory tract infections versus weekly climatic covariates

511 Left panel: time series of weekly URI and LRI incidence and weekly average climatic covariates.

512 Right panel: Scatter plots with fitted lines and 95% confidence interval from univariate Poisson
513 generalized linear model for weekly URI or LRI incidence vs. each climatic covariate.

514

515

516

517

For peer review only

For peer review only - http://bmjopen.bmj.com/site/about/guidelines.xhtml

518 **Supplementary Figures**

519 **Figure S1.** Maps for empirical Bayesian estimated incidence rates of acute respiratory tract infections by year at ward level

521 District labels are shown. 1-12: district 1-12; 13-24: district Binh Chanh, Binh Tan, Binh Thanh, Can Gio, Cu Chi, Go Vap, Hoc Mon, Nha Be, Phu Nhuan, Tan Binh, Tan Phu, Thu Duc respectively.

523 Locations of study hospitals are shown in green. *Rural districts.

525 **Figure S2.** Maps for empirical Bayesian estimated incidence rates of upper respiratory tract infections and lower respiratory tract infections at ward level

527 a) Empirical Bayesian estimated incidence rates (EBR) per 1,000 population of URIs with locations of hospitals in green, b) EBR of LRIs per 1000 population with locations of hospitals in green. CH1: Children Hospital 1, CH2: Children Hospital 2; HTD: Hospital for Tropical Diseases. District names and district boundaries (grey) are shown. *Rural districts.

532 **Figure S3.** Diagnostic plots of the full-generalized additive mixed models (GAMM) for upper respiratory tract infections and lower respiratory tract infections

534 Left panel: URIs; right panel: LRIs.

536 **Figure S4.** Autocorrelation function and partial autocorrelation function of the residuals of the full generalized additive mixed models (GAMM) for upper respiratory tract infections and lower respiratory tract infections (LRIs)

539 Left panel: URIs; right panel: LRIs.

For peer review only

For peer review only - http://bmjopen.bmj.com/site/about/guidelines.xhtml

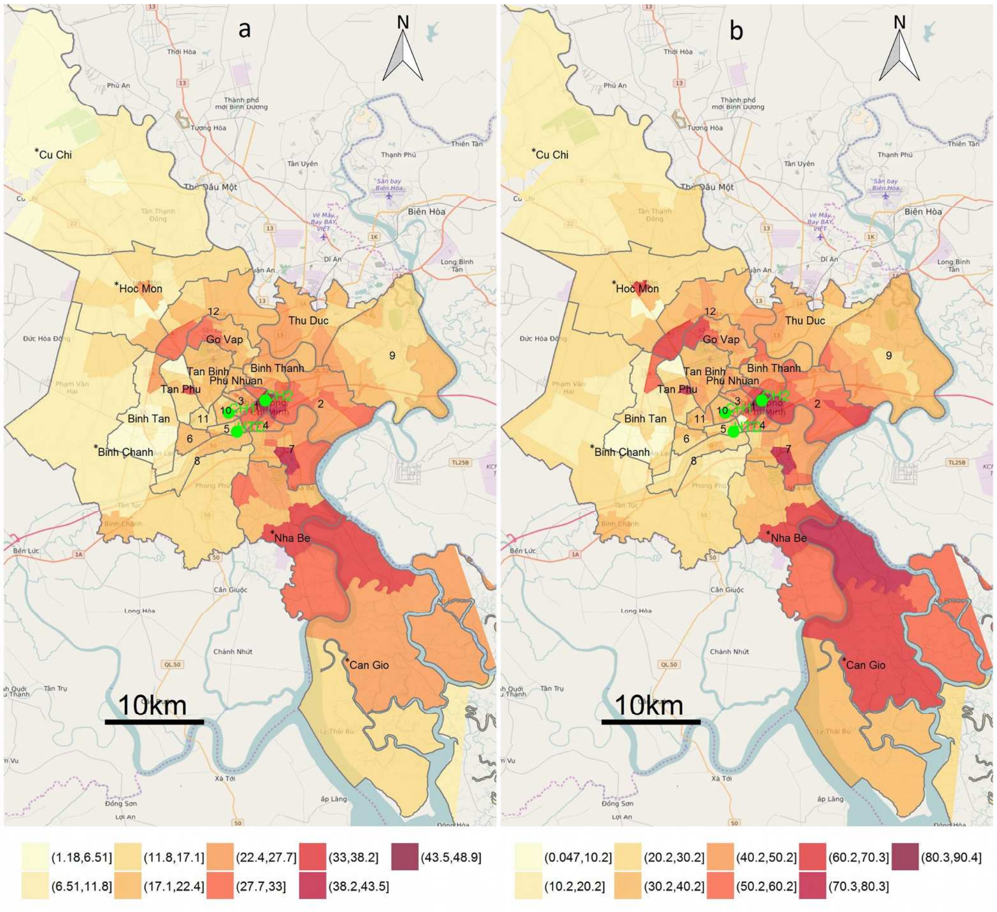

Figure 1

180x164mm (300 x 300 DPI)

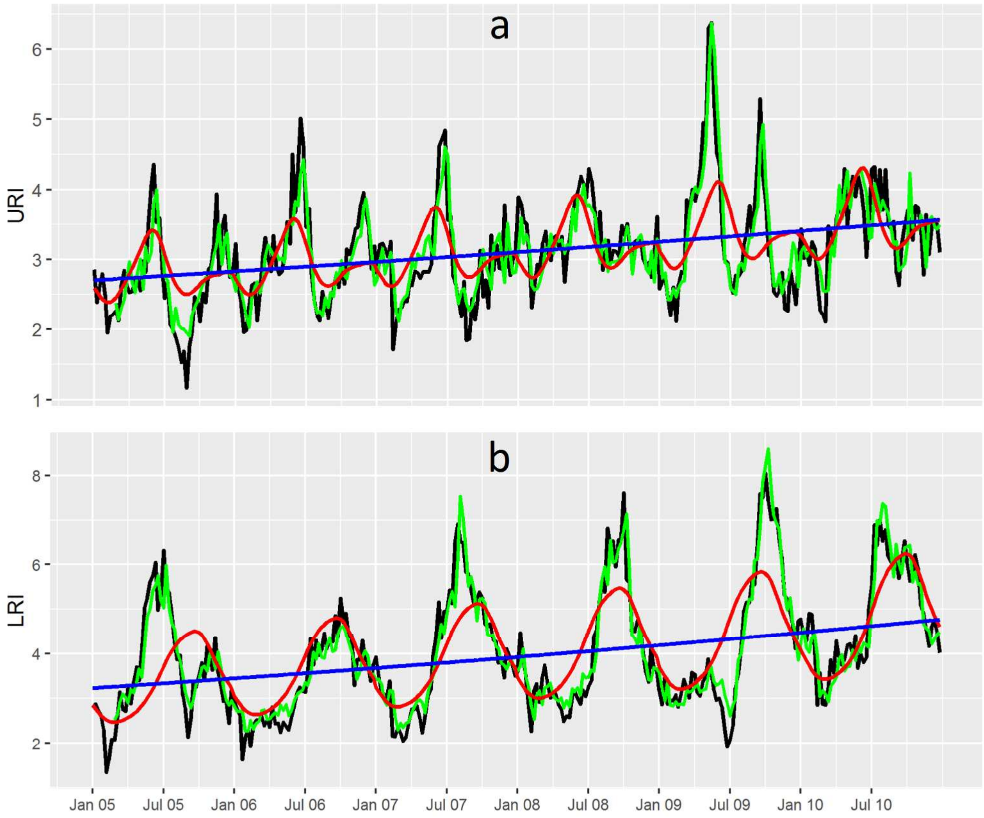

Figure 2

177x152mm (300 x 300 DPI)

only

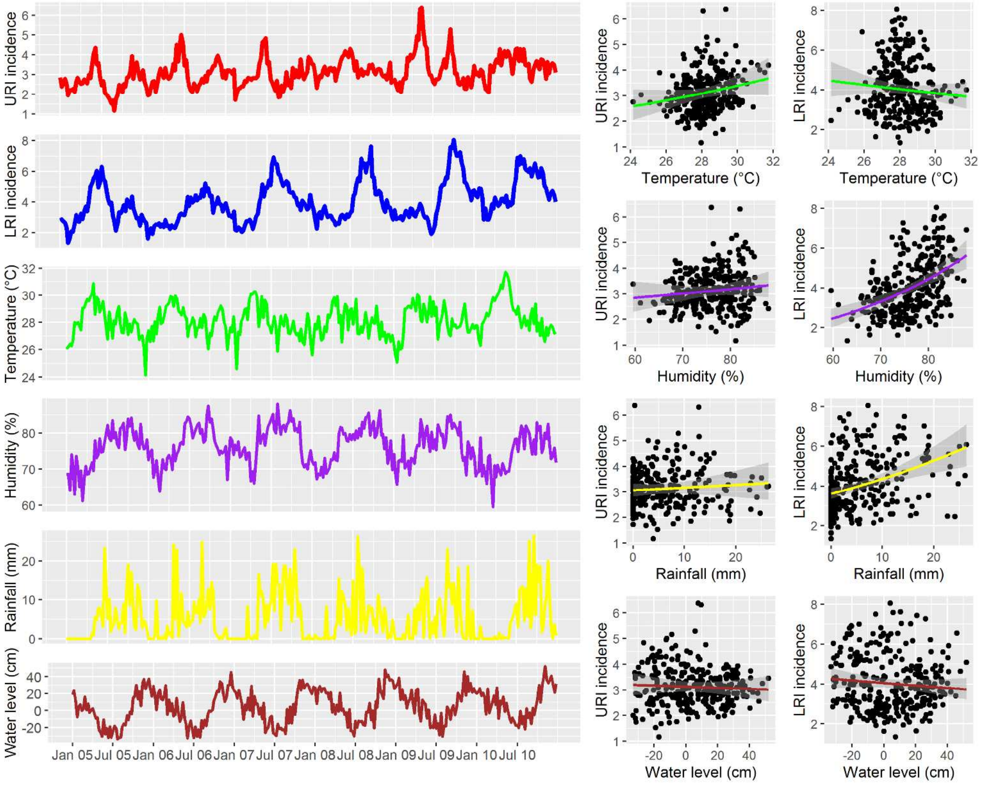

Figure 3

256x203mm (300 x 300 DPI)

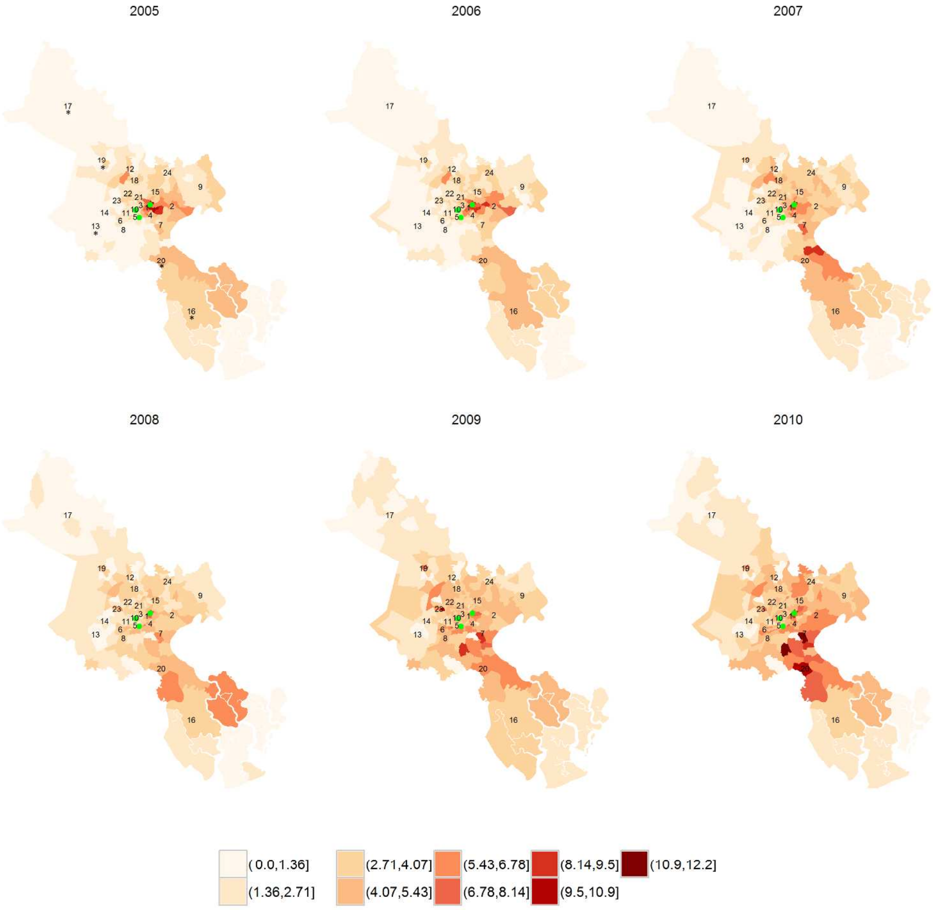

Figure S1

254x254mm (300 x 300 DPI)

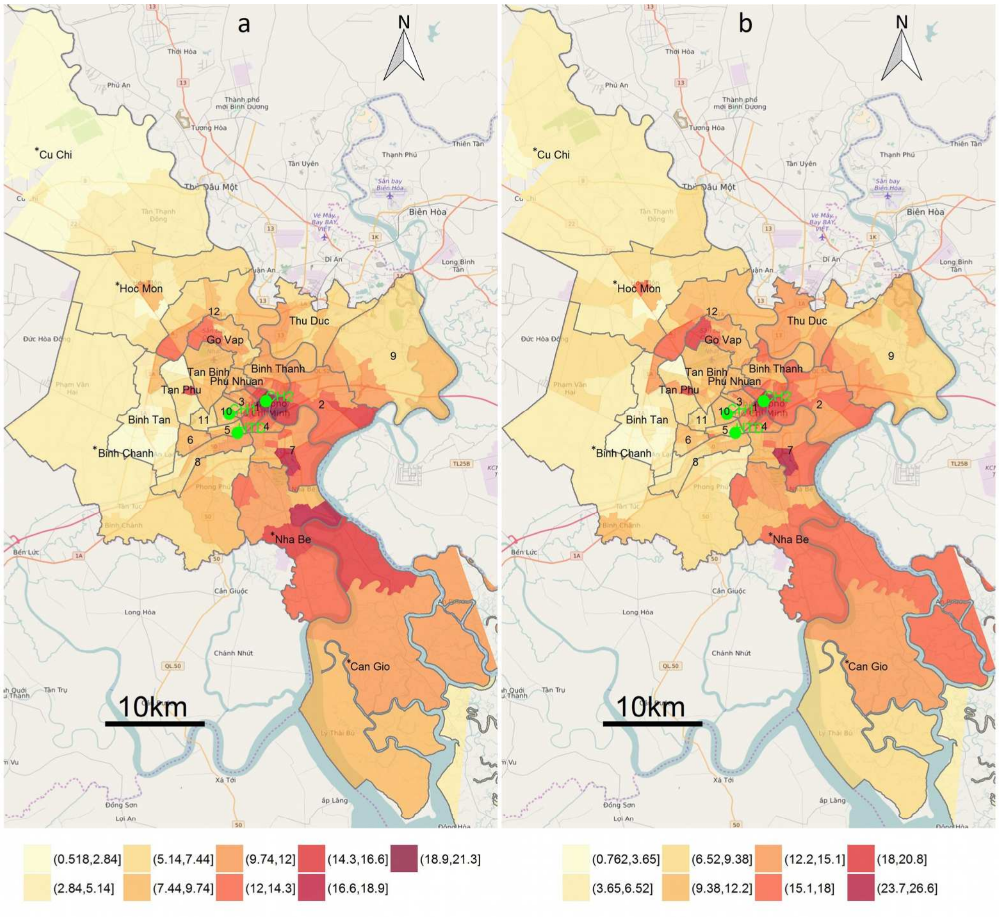

Figure S2

180x164mm (300 x 300 DPI)

For peer review only - http://bmjopen.bmj.com/site/about/guidelines.xhtml

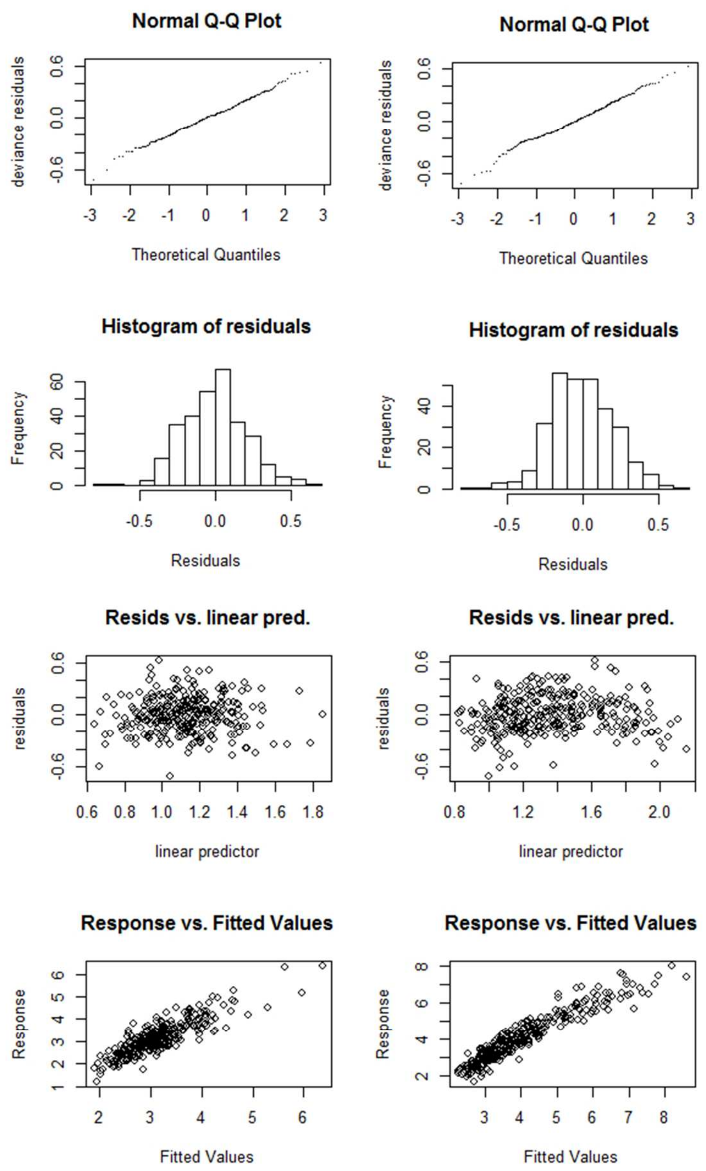

Figure S3

49x83mm (300 x 300 DPI)

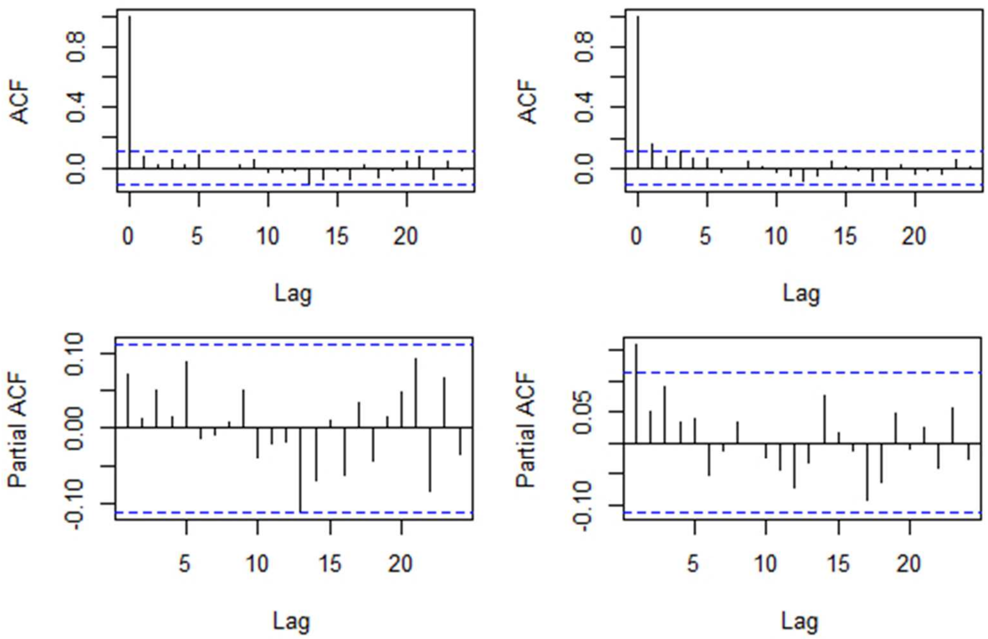

Figure S4

47x33mm (300 x 300 DPI)

BMJ Open

A retrospective analysis assessing the spatial and temporal distribution of pediatric acute respiratory tract infections in Ho Chi Minh City, Vietnam

Journal:	*BMJ Open*
Manuscript ID	bmjopen-2017-016349.R2
Article Type:	Research
Date Submitted by the Author:	09-Nov-2017
Complete List of Authors:	Ho, Nhan Thi; Oxford University Clinical Research Unit, Enteric infections<br>Thompson, Corinne; Oxford University Clinical Research Unit, Enteric infections<br>Nhan, Le Nguyen Thanh; Children's hospital one<br>Van, Hoang Minh Tu; Children's hospital one<br>Dung, Nguyen Thanh; The Hospital for Tropical Diseases<br>Tran My, Phuc; Oxford University Clinical Research Unit, Enteric infections<br>Quang, Vo Minh; The Hospital for Tropical Diseases<br>Minh, Ngo Ngoc Quang; The Hospital for Tropical Diseases<br>Tuan, Tran Anh; Oxford University Clinical Research Unit, Enteric infections<br>Hung, Nguyen Thanh; Children's hospital one<br>Tuan, Ha Manh ; Children's hospital 2<br>Vinh Chau, Nguyen Van; The Hospital for Tropical Diseases<br>Wolbers, Marcel; Oxford University Clinical Research Unit, Enteric infections<br>Thwaites, Guy; Oxford University Clinical Research Unit, Enteric infections<br>Choisy, Marc; Oxford University Clinical Research Unit, Enteric infections<br>Baker, Stephen; Oxford University Clinical Research Unit, Enteric infections
**Primary Subject Heading**:	Respiratory medicine
Secondary Subject Heading:	Epidemiology, Infectious diseases, Paediatrics
Keywords:	Acute respiratory infections, upper respiratory infections, lower respiratory infections, spatial distribution, temporal patterns, pediatrics

SCHOLARONE™
Manuscripts

A retrospective analysis assessing the spatial and temporal distribution of pediatric acute respiratory tract infections in Ho Chi Minh City, Vietnam

Nhan Thi Ho [1†#], Corinne Thompson [1,2,3†], Le Nguyen Thanh Nhan [4], Hoang Minh Tu Van [5], Nguyen Thanh Dung [6], Tran My Phuc [1], Vo Minh Quang [6], Ngo Ngoc Quang Minh [4], Tran Anh Tuan [4], Nguyen Thanh Hung [4], Ha Manh Tuan [5], Nguyen Van Vinh Chau [6], Marcel Wolbers [1,2], Guy E Thwaites [1,2], Marc Choisy [7] and Stephen Baker [1,2,8*]

[1] Oxford University Clinical Research Unit, Wellcome Trust Major Overseas Program, Ho Chi Minh City, Vietnam

[2] Centre for Tropical Medicine and Global Health, Nuffield Department of Medicine, University of Oxford, United Kingdom

[3] The London School of Hygiene & Tropical Medicine, United Kingdom

[4] Children's Hospital 1, Ho Chi Minh City, Vietnam

[5] Children's Hospital 2, Ho Chi Minh City, Vietnam

[6] Hospital for Tropical Diseases, Ho Chi Minh City, Vietnam

[7] Institute of Research and Development, Vietnam

[8] The department of Medicine, the University of Cambridge, Cambridge, United Kingdom

† These authors contributed equally to this work.

Current institution: Columbia University Medical Center, New York, United States

* Corresponding Author: Prof. Stephen Baker, Oxford University Clinical Research Unit, 764 Vo Van Kiet, Quan 5, Ho Chi Minh City, Vietnam. Tel: +84 89241761 Fax: +84 89238904 sbaker@oucru.org

Author email addresses: Nhan Thi Ho: nth2111@cumc.columbia.edu, Corinne Thompson: corinne.thompson1@gmail.com, Le Nguyen Thanh Nhan: nhanlnt@oucru.org, Hoang Minh Tu Van: vanhmt@oucru.org, Nguyen Thanh Dung: bsdungbvbnd@gmail.com, Tran My Phuc:

For peer review only - http://bmjopen.bmj.com/site/about/guidelines.xhtml

phuctm@oucru.org, Vo Minh Quang: bsvominhquang@gmail.com, Ngo Ngoc Quang Minh: minhnnq@yahoo.com, Tran Anh Tuan: drtat@hotmail.com, Nguyen Thanh Hung: drthanhhung@gmail.com, Ha Manh Tuan: manhtuanzu@yahoo.com, Nguyen Van Vinh Chau: chaunvv@oucru.org, Guy Thwaites: gthwaites@oucru.org, Marc Choisy: mchoisy@oucru.org, Marcel Wolbers: marcel.wolbers@gmx.ch, Stephen Baker: sbaker@oucru.org

For peer review only

For peer review only - http://bmjopen.bmj.com/site/about/guidelines.xhtml

Abstract

Background: Acute respiratory infections (ARIs) are the leading cause of morbidity and mortality in young children in developing countries. Using routine hospital data we aimed to examine the spatial distribution, temporal trends, and climatic risk factors of pediatric ARIs in Vietnam.

Methods: Data from hospitalized pediatric (<16 years old) patients with ARIs residing in Ho Chi Minh City (HCMC) between 2005-2010 were retrieved from the two main Children's Hospitals and the Hospital for Tropical Diseases in HCMC. Spatial mapping and time series analysis were performed after disaggregating data into upper respiratory infections (URIs) and lower respiratory infections (LRIs).

Results: Over the study period, there were 155,999 pediatric patients admitted with ARIs (33% of all hospital admissions). There were 68,120 URIs (14%) and 87,879 LRIs (19%). The most common diagnoses were acute pharyngitis (28% of all ARI), pneumonia (21%), bronchitis (18%), and bronchiolitis (16%). A significant increasing trend over time was found for both URIs (mean weekly incidence per 1,000 population (I)=3.12, incidence rate ratio for 1 week increase in time (RR)[95%CI]=1.09[1.02,1.17]) for URI and (I=4.02, RR[95%CI]=1.08[1.006,1.16]) for LRI. The weekly URI incidence peaked in May-June and was significantly associated with lags in weekly URI incidence and the average humidity, rainfall, and water level. The weekly LRI incidence exhibited significant seasonality ($p<0.0001$), with an annual peak in September-October and was significantly associated with lags in weekly LRI incidence and lags in weekly average temperature, rainfall, and water level.

Conclusions: ARIs are a leading cause of childhood hospitalization in HCMC, Vietnam. The incidence of ARIs was higher in the wet season and higher in specific HCMC districts. These results may guide health authorities in where and when to effectively allocate resources for the prevention and control of ARIs.

For peer review only

For peer review only - http://bmjopen.bmj.com/site/about/guidelines.xhtml

Strengths and limitations of the study

- Using routine data from electronic hospital databases we were able to retrieve (based on International Classification of Disease version 10 (ICD10) codes) access information on pediatric admissions due to acute respiratory infections (ARIs) over 6 year period in Ho Chi Minh City to examine the spatial distribution, temporal patterns, and climatic risk factors of pediatric ARIs.
- Pediatric ARI admissions were mapped and compared with all other pediatric admissions to assess the spatial distribution of ARI admissions in comparison with the spatial referral pattern of the study hospitals.
- Data were stratified in two clinically differentiable groups: upper respiratory infections (URIs) and lower respiratory infections (LRIs) to utilize the general ICD10 codes in the databases.
- Our findings were generated using hospitalized patient data and thus might not be generalizable for all outpatients. This may be particularly true for URIs, as many patients with URIs do not require hospitalization.
- There was limited data on the specific pathogens associated with ARIs in the databases. URIs and LRIs are comprised of various diseases associated with a wide range of pathogens, which may have different spatial temporal patterns.

Key words

Acute respiratory infections, upper respiratory infections, lower respiratory infections, spatial distribution, temporal patterns, pediatrics

For peer review only

Introduction

Acute respiratory tract infections (ARIs) are comprised of upper respiratory tract infections (URIs) and lower respiratory tract infections (LRIs). URIs are associated with infections at or above the larynx, are most commonly induced by viruses and include rhinitis (the common cold), sinusitis, ear infections, acute pharyngitis, epiglottitis, and laryngitis. LRIs are comprised of bacterial or viral infections occurring below the larynx and encompass bronchitis, bronchiolitis, and pneumonia [1]. ARIs are the leading cause of morbidity and mortality in young children [2–4], accounting for >18% of all childhood deaths globally [5]. In 2010, there were approximately 120 million episodes and 1.3 million deaths associated with pneumonia worldwide, the vast majority of these occurred in Southeast Asia and sub-Saharan Africa [5,6]. In industrializing regions, ARIs in children aged <5 years are generally more severe than in older children and adults, resulting in a higher case-fatality than in industrialized countries [1,7]. This disparity in mortality rate is multifactorial and associated with differing etiological agents, available therapies, exposure frequencies, and host immunity.

In Ho Chi Minh City (HCMC), Vietnam, ARIs are amongst the most common cause of childhood hospitalizations [8–10]. The common identified pathogens of hospitalized ARI in HCMC include respiratory syncytial virus (RSV), rhinovirus, coronavirus, influenza, and bocavirus [8–10], in addition to the bacterial pathogens *Streptococcus pneumoniae* and *Haemophilus influenzae* type b (Hib) [11,12]. With limited access to costly vaccines for bacterial etiologies and a lack of vaccines for viral causes of ARIs, a greater understanding of the risk factors and sub-populations at risk of ARIs in HCMC is necessary to effectively focus existing prevention measures such as the encouragement of breastfeeding, hand washing with soap, and a reduction in household air pollution [2].

There are data to suggest that the incidence of ARIs is associated with specific spatial, meteorological, and socio-demographic risk factors [13–16]. However, specific temporal and climatic risk factors for both URIs and LRIs in HCMC have not been investigated. Here we aimed to examine the spatial

For peer review only - http://bmjopen.bmj.com/site/about/guidelines.xhtml

distribution, temporal patterns, and climatic risk factors for hospitalized pediatric ARIs in HCMC. This analysis was performed to generate a better understanding of the factors that may influence ARIs and in order to guide healthcare resources for this common pediatric disease syndrome.

Methods

Data sources

Data for this study was derived from the electronic hospital databases of all pediatric (<16 years) inpatient admissions to three large tertiary hospitals located in central HCMC: Children's Hospital 1 (CH1), Children's Hospital 2 (CH2), and the Hospital for Tropical Diseases (HTD). Data from the two children hospitals were collected between 2005 and 2010 (inclusive) while the data from HTD were available from 2008 to 2010 only.

ARIs are caused by a range of differing pathogens and may have different spatiotemporal patterns and associations with climate and other covariates. Therefore, we allocated respiratory infections into two major clinically differentiable groups based on the International Classification of Disease version 10 (ICD10) codes version 2016 outlined by the World Health Organization (WHO): 1) URIs were amalgamated under the ICD10 codes J00:J06 and J9:J11 for influenza like illnesses and other acute upper respiratory infections and 2) LRIs, which incorporated the ICD10 codes J12:J18 and J20:J22 for pneumonia and other acute lower respiratory infections. For the purposes of this study the data from patients with the selected ICD10 codes who resided in HCMC were extracted and analyzed. Each patient record included data regarding age, sex, date of admission and discharge, ICD10 code at discharge, and residential address.

HCMC is a large economic center in the South of Vietnam with a population of >8 million people. Geographically, it is divided into 24 districts (land areas ranging from 4.18 km^2- 704.22 km^2), which are subdivided in 322 smaller administrative wards. There are 5 rural districts (population density ranging

For peer review only - http://bmjopen.bmj.com/site/about/guidelines.xhtml

from 100 persons/km^2 to 3,326 persons/km^2) and 19 urban districts (population density ranging from 2,360 persons/km^2 to 45,582 persons/km^2). There are at least one local (district) hospital in each district, which serves both outpatients and inpatients of all ages. However, most local hospitals, especially those in urban districts receive mostly adult outpatients. There are two central hospitals (CH1 and CH2) that specialize in serving the pediatric population in HCMC. Another further central hospital (HTD) specializes in infectious diseases and also receives pediatric patients. While pediatric outpatients in HCMC may choose to visit local or private healthcare facilities or tertiary hospitals, most pediatric patients requiring hospitalization in HCMC are referred or self-referred to one of the three study hospitals. Therefore, the three study hospitals (CH1, CH2, and HTD) receive the majority of pediatric inpatients from HCMC.

There are two main seasons in HCMC: a dry season from December to April and a rainy season from May to November. Population and district characteristic data (urban vs. rural districts, district population density, district economic indicators (expenditure of district budget, revenues of district budget), district agricultural indicators (number of pigs in herds > 2 months of age by district, % area of district used as rice paddy), and district education indicator (number of primary and secondary schools per district) were obtained from the HCMC Statistical Office [17]. As population figures for those aged <16 years per ward or district was not obtainable, the total population was used to estimate disease incidence. Patient addresses in HCMC were geocoded to districts and wards. City level weekly average climate data during 2005–2010 were obtained from the Ministry of Natural Resources and Environment of Vietnam including relative humidity (median=76.5%, range=61.7% to 88%), temperature (median=28.1°C, range=24.4°C to 31.8°C), rainfall (median=24.4mm, range=0mm to 230.7mm) and water level of the Dong Dien River (median=4.93cm, range= -34.43cm to 49.43cm) [18].

Spatial mapping

For peer review only - http://bmjopen.bmj.com/site/about/guidelines.xhtml

182 The variance of raw ward ARI incidence rates (number of ARI cases per 1,000 ward total population for the whole study period) may largely vary due to the large variation in ward population size in HCMC. To take into account the spatial heterogeneity of variance and any dependence of ARI incidence rates between wards, ward ARI incidence rates were smoothed using local empirical Bayes estimates for rates reduced to a neighborhood mean [19]. The neighborhoods were selected using the neighborhood list based on wards with contiguous boundaries (R package spdep version 0.5-88) [20]. Kulldorff and Nagarwalla's method over the centroids was be used to scan for clusters of ARIs [21]. This method allows the detection of possible cluster of any size, at any location in a population with inhomogeneous spatial density of HCMC. A Poisson generalized linear model was used to explore the relationship between district ARI incidence and district characteristics. Confidence intervals and tests were based on robust standard errors to control for mild violation of the distributional assumption that the variance equals the mean [22].

Time series analysis

Citywide ARI weekly incidences (number of cases per 1,000 population) were examined for seasonality, time trends and associations with climatic covariates by Poisson generalized additive mixed models (GAMM) implemented in the R package mgcv version 1.8-7 [23,24]. The seasonal cycles and time trends of weekly ARI incidence were evaluated by the basic GAMM models containing the week of year (Ws=1 to 52)) as cyclic cubic regression splines and the week of the whole study period (Wt=1 to 312)) as cubic regression spline. As weekly ARI incidence may be associated with the ARI incidence and climatic condition of the previous weeks, in addition to the elements in the basic model, the full GAMM model contained lags of ARI weekly incidence up to eight weeks, concurrent climatic condition and lags of climatic condition up to 8 weeks. The performance of the GAMM models was evaluated using adjusted R-squared and model diagnostic plots. Temporal correlation of the model residuals was evaluated by autocorrelation function (ACF) and partial autocorrelation function (PACF).

For peer review only - http://bmjopen.bmj.com/site/about/guidelines.xhtml

All analyses and results were based on the records with available relevant information (complete cases). Spatial mapping and statistical analyses were stratified by URIs and LRIs and all analyses were performed in R version 3.2.2 [25].

Results

Basic features of acute respiratory infections in the pediatric population of Ho Chi Minh City

From 2005 to 2010, there were 155,999 children (<16 years old) residing in HCMC admitted to the three study hospitals for ARIs (33% of all admissions); 323,245 patients were admitted for conditions other than ARIs. There were 68,120 patients with ICD10 codes synonymous with acute URIs (14% of all admissions) and 87,879 patients with ICD10 codes associated with acute LRIs (19% of all admissions). The proportion of males (62%) and females (38%) was comparable between URIs and LRIs. The median age of the patients was 1.5 years (interquartile range [IQR]: 0.7-2.9 years). However, patients with LRIs were significantly younger than those with URIs (median age in years (IQR) = 1.1 (0.5, 2.1) vs. 2.1 (1.1, 3.9)), Wilcoxon's test $p<0.0001$) and LRI patients were hospitalized significantly longer than those with URIs (median days of hospitalization (IQR) = 6.0 (4.0, 9.0) vs. 4.0 (2.0, 5.0), Wilcoxon's test $p<0.0001$) (Table 1).

Overall, the most common diagnoses were acute pharyngitis (28% of all ARIs), pneumonia (21% of all ARIs), bronchitis (18% of all ARIs) and bronchiolitis (16% of all ARIs). Among the URI patients, the majority of patients were diagnosed with acute pharyngitis (63% of all URIs), acute upper respiratory infections of multiple and unspecified sites (14%), acute tonsillitis (8%), acute laryngitis and tracheitis (6%), and acute nasopharyngitis (6%). Among the LRI patients, the most common diagnoses were pneumonia with an unspecified organism (37% of all LRIs), acute bronchitis (32%), acute bronchiolitis (29%), and bacterial pneumonia not elsewhere classified (2%) (Table 1).

For peer review only - http://bmjopen.bmj.com/site/about/guidelines.xhtml

For peer review only

The spatial distribution of pediatric acute respiratory infections in Ho Chi Minh City

The raw ward-level incidence rates of ARIs for the entire study period ranged from zero to 50 per 1,000 total population (median (IQR) = 18.8 (14.2, 23.5)) and the smoothed ward level empirical Bayesian estimated ARI incidence rates ranged from 1.2 to 48.8 ARI cases per 1,000 population (median (IQR) = 18.6 (14.7, 23.7)). High incidence rates (>30 cases/1,000 population) of ARI were observed not only in the wards surrounding the three study hospitals (wards in districts 1 and 4 in central HCMC) but also in multiple wards located outside the central region of the city, specifically wards located in districts 2, 7, and Nha Be (observed vs. expected incidence rate ratio >1.4, $p<0.0001$ using Kulldorff and Nagarwalla's scan) (Figure 1a). Notably, the spatial distribution of ARIs and diseases other than ARI were comparable (Figure 1a and 1b). We observed an increase with time in ward incidence of ARIs in the majority of the wards with the most apparent high incidences in wards in the south of the city (Figure S1). We additionally found the spatial distribution of URIs and LRIs were comparable (Figure S2).

The district level incidence over the entire study period of ARIs was significantly lower in the districts located further away from the hospitals (mean district incidence=26.7 per 1,000 population, RR for 2 times increase in distance to hospital =0.88, 95% CI=[0.80, 0.96]) and in rural districts (RR for urban vs. rural districts =1.54, 95% CI=[1.10, 2.15]) (Figure 1a). Other district characteristics were not significantly associated with district ARI incidence.

For peer review only

Temporal patterns and climatic risk factors of pediatric acute respiratory infections

The mean of weekly incidence of URIs for the study period was 3.12/1,000 population. Weekly URI incidence showed a relatively small increase with time, with a difference in mean weekly URI incidence at the end and the start of the study period of 0.86 patients per 1,000 population. The basic Poisson GAMM model indicated that URI admissions in the study hospitals exhibited a significant increasing trend with time ($p<0.0013$) and seasonality ($p<0.0001$) with a peak in May to June and a smaller peak in December to January (Figure 2a).

For peer review only - http://bmjopen.bmj.com/site/about/guidelines.xhtml

The mean weekly LRI incidence for the study period was 4.02/1,000 population. There was a significant increase of weekly LRI incidence over time (p=0.0037) with a substantial difference in the mean weekly LRI incidence at the end vs. the start of the study period of 1.54 patients/1,000 population. The seasonal cycle of LRIs (p<0.0001) was more obvious than that of URIs, with a single, wide annual peak in the months of September and October (Figure 2b).

In a univariate analysis with Poisson generalized linear model, we found that weekly URI incidence was significantly associated with weekly average temperature (RR for 1 degree increase of temperature=1.048, 95%CI=[1.030,1.066]) and humidity (RR for 1% increase in relative humidity=1.006, 95%CI= [1.001,1.011]). Further, weekly LRI incidence was significantly associated with weekly average humidity (RR=1.030, 95%CI=[1.024,1.036]) and rainfall (RR for 1mm increase in rainfall= 1.019, 95%CI=[1.014,1.024]) (Figure 3). However, after controlling for seasonality, time trend, lags of weekly incidence, and other climatic covariates in the full GAMM model, the weekly URI incidence was significantly associated with one and two week lags in URI incidence (RR=1.202, 95%CI=[1.159,1.245] and RR=1.063, 95%CI=[1.019,1.109], respectively), one and three week lags in weekly average humidity (RR=1.009, 95%CI=[1.003,1.014] and RR=0.990, 95%CI=[0.984,0.996], respectively), a three week lag in weekly average rainfall (RR=1.006, 95%CI=[1.003,1.009]) and a four week lag in weekly average river water level (RR for 1 cm increase in water level=1.003, 95%CI=[1.002,1.005]) (Table 2).

After adjusting for other factors in the full GAMM model, weekly LRI incidence was significantly associated with a one and two week lags in LRI incidence (RR=1.173, 95%CI=[1.138,1.208] and RR=1.053, 95%CI=[1.015,1.092] respectively), a one week lag in weekly average temperature (RR=0.976, 95%CI=[0.953,0.998], a one week lag in weekly average rainfall (RR=1.004, 95%CI=[1.001,1.007], and a one week lag in weekly average water level (RR=0.998, 95%CI=[0.997,1.000] (Table 2). The time trends remained significant for both URIs (incidence rate ratio

For peer review only - http://bmjopen.bmj.com/site/about/guidelines.xhtml

for 1 week increase in time (RR)=1.09, 95%CI=[1.02,1.17], p=0.0096) and LRIs (RR=1.08, 95%CI=[1.006,1.16], p=0.0345) while only LRIs exhibited significant seasonality (p<0.0001) in the full GAMM models. The full GAMM models fitted the data well for both URIs (adjusted R squared =0.72) and LRIs (adjusted R squared =0.88) (Figure 2 and Figure S3). Temporal correlation of weekly incidence was well controlled for URIs and also relatively well controlled for LRIs in the full GAMM models (Figure S4).

Discussion

Our study examined the spatial distribution, temporal patterns, and climatic risk factors of ARIs in HCMC, Vietnam. The findings were based on the data of a large number of ARI inpatients retrieved from routinely collected electronic hospital databases over a 6-year period. Our data showed that ARIs accounted for one third of all hospital admissions over the study period and were the most common cause of hospitalized diseases in children in HCMC. The spatial distribution of ARIs was comparable to the spatial referral patterns of study hospitals in HCMC (the spatial distribution of diseases other than ARIs). High incidence of ARIs was found not only in districts close to the study hospitals (districts 1 and 4) but also districts 2, 7, and Nha Be, which are located further away from three study hospitals. This finding is likely a result of urbanization or better economic status of those living in these districts.

The observed increase in hospitalized ARI incidence with time may be explained by better healthcare awareness within the population, through better access to tertiary hospitals, better healthcare policies (free healthcare for children aged <6 years was initiated in Vietnam), or a real increase in ARI incidence. The citywide increase of ARI incidence, especially LRIs, may also be attributed to an increase in air pollution. Pollution was shown to be associated with an increase in LRI incidence in study conducted by the Collaborative Working Group on Air Pollution, Poverty, and Health in HCMC [26]. The number of beds in these tertiary hospitals did not increase over the study period, and the policy of these hospitals is to accommodate all patients visiting the hospitals regardless of the capacity of the hospital and staff. An

For peer review only - http://bmjopen.bmj.com/site/about/guidelines.xhtml

increase in ARI admission indicates an increase in burden for these tertiary hospitals and an increased workload for healthcare, which may impact negatively on the quality of care. Therefore, there should be effective policies to improve the allocation of healthcare resources in these hospitals as well as to improve the quality and capacity of local healthcare facilities to share this increasing disease burden.

The weekly incidence of both URIs and LRIs showed significant association with 1 and 2 week lags of incidence indicating that temporal correlation might play an important role in URI and LRI incidence. Furthermore, we additionally noted a difference in seasonal patterns of URIs (two small peaks a year) and LRIs (one strong peak a year) and a difference in association patterns with climatic factors of URIs and LRIs. These findings suggested that climatic factors might differentially influence the pathogens causing URIs and LRIs in HCMC. The association of URI incidence with one and three week lags of weekly average humidity was consistent with the association between the seasonality of absolute humidity and influenza-like illness (ILI) in Vietnam as previously reported [27]. In addition, the national surveillance of influenza and ILI in Vietnam during the study period exhibited a similar seasonal pattern with two peaks a year at approximately the same months with the two peaks of URIs in our data [28]. The exceptional high peak of URIs without a concurrent peak of LRIs in 2009 also occurred simultaneously with 2009 H1N1. Therefore, influenza and ILI were likely key contributors to the seasonality and the peaks of URIs in our data. Moreover, a study conducted at CH2 investigating viral ARI pathogens found that the peak of RSV infections occurred in rainy season (May-November in HCMC). This period included the peak of LRI months and the larger URI peak [10]. Furthermore, RSV is also thought to predispose infected hosts to bacterial infections [29]. In addition, a sharp increase of LRI incidence was observed soon after the peak of URIs (May-June) suggests that URIs and LRIs share some common epidemiological features. As a consequence, appropriate treatment and transmission control for URIs may impact on the incidence of later hospitalization due to LRIs. As the peaks of hospitalization for LRIs are in September-October, resources for pediatric healthcare should be prepared and local healthcare facilities should be enhanced to share the increase in hospitalization caused by LRIs at tertiary hospitals during this period.

For peer review only - http://bmjopen.bmj.com/site/about/guidelines.xhtml

A large proportion of ARI admissions in these data were URIs, two third of these were diagnosed with acute pharyngitis. These conditions can usually be treated in outpatient settings and do not typically require hospitalization. However, the decision to hospitalize ARI patients in these tertiary hospitals is not strictly based on the WHO Integrated Management of Childhood Illness (IMCI) guidelines and is also dependent on the preferences of caregivers and the subjective patient evaluation of the attending physicians. Therefore, there should stricter policies for applying IMCI guidelines and other clinical criteria to reduce unnecessary URI admissions in these hospitals, as well as to improving outpatient care and follow up.

Our study has limitations as the findings were generated using routine data of from hospitalized patients. Therefore, are finding may be generalizable for outpatients, especially for URIs, as many patients with URI may not be admitted to hospital. The findings for LRIs may be more generalizable as LRIs are more severe than URIs and more likely to be hospitalized. Second, the databases contained mostly general ICD10 codes and very limited specific diagnostic information regarding the ARI pathogens. Different pathogens can cause differing URI and LRI disease presentations and different pathogens may be influenced differently by climatic, environmental, population factors as well as host immunity. Therefore, further surveillance and studies investigating different groups of pathogens for ARIs are necessary.

Conclusion

We found that ARIs accounted for one third of total hospital admission and were the leading cause of hospitalization in children in HCMC, Vietnam. The spatial distribution of hospitalized ARIs was similar to the spatial referral patterns of the study hospitals with high incidences in the districts surrounding the locations of the three study hospitals and also in three districts further out the city. The seasonality of ARIs may be explained by the association of ARI incidence with various climatic factors (temperature, humidity and rainfall) and the seasonality of influenza, ILI, and RSV. The increase of LRI incidence soon

For peer review only - http://bmjopen.bmj.com/site/about/guidelines.xhtml

For peer review only

after the peak of URIs suggests that URIs and LRIs share common epidemiological factors or LRIs are complications of URIs. Understanding the spatial distribution, temporal patterns, and climatic risk factors of ARIs should aid the health authorities in outbreak preparation and assist in allocating resources for the prevention and control of the diseases, especially in settings where vaccines for viral and bacterial pathogens of ARIs are not routinely available.

Acknowledgements

The authors are grateful to the staffs of Children's Hospital 1, Children's Hospital 2, and The Hospital for Tropical Diseases for providing the raw databases used in this study.

Footnotes

Contributors

This study was conceptualized by NTH (Nhan Thi Ho), CT, MC and SB. Data was obtained by NTH, CT, LNTN, HMTV, NTD, TMP, VMQ, NNQM, TAT, NTH2 (Nguyen Thanh Hung), HMT and NVVC. Data was analyzed by NTH, CT, MW, MC. NTH, CT and SB wrote the manuscript with input from all authors. NTH, CT, LNTN, HMTV, NTD, TMP, VMQ, NNQM, TAT, NTH2, HMT, NVVC, MW, GET, MC, SB read and approved the final version of the manuscript.

Funding

This work was supported by the Wellcome Trust Vizions strategic award (WT1093824). SB is a Sir Henry Dale Fellow, jointly funded by the Wellcome Trust and the Royal Society (100087/Z/12/Z). NTH is currently funded by Mervyn Susser’s fellowship at Columbia University Medical Center, US. The funders had no role in study design, data collection, data analysis, data interpretation, writing of the report, or decision to submit the article for publication.

For peer review only

For peer review only - http://bmjopen.bmj.com/site/about/guidelines.xhtml

Competing interests

We have read and understood BMJ policy on declaration of interests and declare that we have no competing interests.

Ethical approval

Ethical approval was granted from all of the three study hospitals: Children Hospital 1, Children Hospital 2 and the Hospital for Tropical Diseases.

Data sharing statement

Data will be available upon reasonable request to corresponding author.

References

1. Simoes EAF, Cherian T, Chow J, Shahid-Salles SARL, John TJ. Acute Respiratory Infections in Children. Disease Control Priorities in Developing Countries. 2nd ed. Washington DC: World Bank; 2006.
2. WHO/UNICEF Ending Preventable Child Deaths from Pneumonia and Diarrhoea by 2025: The Integrated Global Action Plan for Pneumonia and Diarrhoea (GAPPD). Geneva; 2013.
3. Lozano R, Naghavi M, Foreman K, Lim S, Shibuya K, Aboyans V, et al. Global and regional mortality from 235 causes of death for 20 age groups in 1990 and 2010: A systematic analysis for the Global Burden of Disease Study 2010. Lancet. 2012;380: 2095–2128. doi:10.1016/S0140-6736(12)61728-0
4. Williams BG, Gouws E, Boschi-Pinto C, Bryce J, Dye C. Estimates of world-wide distribution of child deaths from acute respiratory infections. Lancet Infectious Diseases. 2002. pp. 25–32. doi:10.1016/S1473-3099(01)00170-0
5. Liu L, Johnson HL, Cousens S, Perin J, Scott S, Lawn JE, et al. Global, regional, and national causes of child mortality: An updated systematic analysis for 2010 with time trends since 2000.

For peer review only - http://bmjopen.bmj.com/site/about/guidelines.xhtml

Lancet. 2012;379: 2151–2161. doi:10.1016/S0140-6736(12)60560-1

6. Fischer Walker CL, Rudan I, Liu L, Nair H, Theodoratou E, Bhutta ZA, et al. Global burden of childhood pneumonia and diarrhoea. Lancet. 2013;381: 1405–1416. doi:10.1016/S0140-6736(13)60222-6

7. Global Burden of Disease Pediatrics Collaboration, Kyu HH, Pinho C, Wagner JA, Brown JC, Bertozzi-Villa A, et al. Global and National Burden of Diseases and Injuries Among Children and Adolescents Between 1990 and 2013: Findings From the Global Burden of Disease 2013 Study. JAMA Pediatr. 2016;98121: 1–21. doi:10.1001/jamapediatrics.2015.4276

8. Anders KL, Nguyen HL, Nguyen NM, Thuy NT Van, Van NTH, Hieu NT, et al. Epidemiology and virology of acute respiratory infections during the first year of life: a birth cohort study in Vietnam. Pediatr Infect Dis J. 2015;34: 361–370.

9. Do AHL, van Doorn HR, Nghiem MN, Bryant JE, Hoang TH thi, Do QH, et al. Viral etiologies of acute respiratory infections among hospitalized vietnamese children in Ho Chi Minh City, 2004-2008. PLoS One. 2011;6. doi:10.1371/journal.pone.0018176

10. Tran DN, Trinh QD, Pham NTK, Vu MP, Ha MT, Nguyen TQN, et al. Clinical and epidemiological characteristics of acute respiratory virus infections in Vietnamese children. Epidemiol Infect. 2015; 1–10. doi:10.1017/S095026881500134X

11. Jauneikaite E, Jefferies JM, Hibberd ML, Clarke SC. Prevalence of Streptococcus pneumoniae serotypes causing invasive and non-invasive disease in South East Asia: A review. Vaccine. 2012;30: 3503–3514. doi:10.1016/j.vaccine.2012.03.066

12. Nyambat B, Dang DA, Nguyen HA, Mai TQ, Rani M, Slack MPE, et al. Rapid assessment of Hib disease burden in Vietnam. BMC Public Health. 2011;11: 260. doi:10.1186/1471-2458-11-260

13. du Prel J-B, Puppe W, Gröndahl B, Knuf M, Weigl JAI, Schaaff F, et al. Are meteorological parameters associated with acute respiratory tract infections? Clin Infect Dis. 2009;49: 861–868. doi:10.1086/605435

14. Chen Z, Ji W, Wang Y, Yan Y, Zhu H, Shao X, et al. Epidemiology and associations with climatic

For peer review only

For peer review only - http://bmjopen.bmj.com/site/about/guidelines.xhtml

conditions of Mycoplasma pneumoniae and Chlamydophila pneumoniae infections among Chinese children hospitalized with acute respiratory infections. Ital J Pediatr. 2013;39: 34. doi:10.1186/1824-7288-39-34

15. Welliver R. The relationship of meteorological conditions to the epidemic activity of respiratory syncytial virus. Paediatr Respir Rev. 2009;10: 6–8. doi:10.1016/S1526-0542(09)70004-1

16. Cardoso MRA, Cousens SN, de Góes Siqueira LF, Alves FM, D'Angelo LA V. Crowding: risk factor or protective factor for lower respiratory disease in young children? BMC Public Health. 2004;4: 19. doi:10.1186/1471-2458-4-19

17. Statistical Office in Ho Chi Minh City. Statistical Yearbook of Ho Chi Minh City 2011. Ho Chi Minh City: Ho Chi Minh City Statistical Office; 2012.

18. Loc PD. Ministry of Natural Resources and Environment of the Socialist Republic of Vietnam. Ha Noi; 2013.

19. Cressie N. Smoothing Regional Maps Using Empirical Bayes Predictors. Geogr Anal. 1992;24: 75–95. doi:10.1111/j.1538-4632.1992.tb00253.x

20. Bivand R, Hauke J, Kossowski T. Computing the jacobian in gaussian spatial autoregressive models: An illustrated comparison of available methods. Geogr Anal. 2013;45: 150–179. doi:10.1111/gean.12008

21. Kulldorff M, Nagarwalla N. Spatial disease clusters: detection and inference. Stat Med. 1995;14: 799–810. doi:10.1002/sim.4780140809

22. Cameron AC, Trivedi PK. Microeconometrics using Stata. Stata Press books. 2009;5: 706. doi:10.1016/S0304-4076(00)00050-6

23. Lin X, Zhang D. Inference in generalized additive mixed models by using smoothing splines. J R Stat Soc Ser B. 1999;61: 381–400.

24. Wood SN. Generalized additive models: an introduction with R. Wiley Interdiscip. 2006;16.

25. R Core Team. R: A Language and Environment for Statistical Computing [Internet]. R Foundation for Statistical Computing, Vienna, Austria. 2016. doi:10.1017/CBO9781107415324.004

For peer review only

For peer review only - http://bmjopen.bmj.com/site/about/guidelines.xhtml

467 26. HEI Collaborative Working Group on Air Pollution and Health in Ho Chi Minh City P, Le TG, Ngo L, Mehta S, Do VD, Thach TQ, et al. Effects of short-term exposure to air pollution on hospital admissions of young children for acute lower respiratory infections in Ho Chi Minh City, Vietnam. Res Rep Health Eff Inst. 2012; 5–83.

471 27. Seasonality of absolute humidity explains seasonality of influenza-like illness in Vietnam. Epidemics. Elsevier; 2015;13: 65–73. doi:10.1016/J.EPIDEM.2015.06.002

473 28. Nguyen YT, Graitcer SB, Nguyen TH, Tran DN, Pham TD, Le MTQ, et al. National surveillance for influenza and influenza-like illness in Vietnam, 2006−2010. Vaccine. 2013;31: 4368–4374. doi:10.1016/j.vaccine.2013.07.018

476 29. Beadling C, Slifka MK. How do viral infections predispose patients to bacterial infections? Curr Opin Infect Dis. 2004;17: 185–191. doi:10.1097/00001432-200406000-00003

For peer review only

For peer review only - http://bmjopen.bmj.com/site/about/guidelines.xhtml

Tables

Table 1. The characteristics and diagnoses for all patients with acute respiratory tract infections upon admission

Characteristic	All ARI patients (N=155,999)	URI (N=68,120)	LRI (N=87,879)	p-value
Male sex [a]	96,930/155,997 (62%)	42,251/68,119 (62%)	54,679/87,878 (62%)	0.43*
Age (year) [b]	1.5(0.7,2.9)	2.1(1.1,3.9)	1.1(0.5,2.1)	<0.0001#
Days in hospital [c]	5.0(3.0,7.0)	4.0(2.0,5.0)	6.0(4.0,9.0)	<0.0001#
**ICD10 code at discharge**				
Acute bronchiolitis (J21)	25,123/155,999 (16%)	0/68,120 (0%)	25,123/87,879 (29%)	
Acute bronchitis (J20)	28,202/155,999 (18%)	0/68,120 (0%)	28,202/87,879 (32%)	
Acute laryngitis & tracheitis (J04)	4,323/155,999 (3%)	4,323/68,120 (6%)	0/87,879 (0%)	
Acute nasopharyngitis (J00)	4,049/155,999 (3%)	4,049/68,120 (6%)	0/87,879 (0%)	
Acute obstructive laryngitis & epiglottitis (J05)	9/155,999 (0%)	9/68,120 (0%)	0/87,879 (0%)	
Acute pharyngitis (J02)	43,148/155,999 (28%)	43,148/68,120 (63%)	0/87,879 (0%)	
Acute sinusitis (J01)	592/155,999 (0%)	592/68,120 (1%)	0/87,879 (0%)	
Acute tonsillitis (J03)	5,123/155,999 (3%)	5,123/68,120 (8%)	0/87,879 (0%)	
Acute upper respiratory infections of multiple & unspecified sites (J06)	9,300/155,999 (6%)	9,300/68,120 (14%)	0/87,879 (0%)	
Bacterial pneumonia, not elsewhere classified (J15)	2,016/155,999 (1%)	0/68,120 (0%)	2,016/87,879 (2%)	
Influenza due to other identified virus (J10)	381/155,999 (0%)	381/68,120 (1%)	0/87,879 (0%)	
Influenza, virus not identified (J11)	1,195/155,999 (1%)	1,195/68,120 (2%)	0/87,879 (0%)	
Pneumonia due to *Haemophilus influenza* (J14)	11/155,999 (0%)	0/68,120 (0%)	11/87,879 (0%)	
Pneumonia due to other infectious organisms, not elsewhere classified (J16)	26/155,999 (0%)	0/68,120 (0%)	26/87,879 (0%)	
Pneumonia due to *Streptococcus pneumonia* (J13)	16/155,999 (0%)	0/68,120 (0%)	16/87,879 (0%)	
Pneumonia in diseases classified elsewhere (J17)	1/155,999 (0%)	0/68,120 (0%)	1/87,879 (0%)	
Pneumonia, organism unspecified (J18)	32,371/155,999 (21%)	0/681,20 (0%)	32,371/87,879 (37%)	
Unspecified acute lower respiratory infection (J22)	7/155,999 (0%)	0/68,120 (0%)	7/87,879 (0%)	
Viral pneumonia, not elsewhere classified (J12)	106/155,999 (0%)	0/68,20 (0%)	106/87,879 (0%)	

a: data from 155997 patients; b: data from 147,420 patients; c: data from 96,362 patients.*Chi-square test comparing URIs vs. LRIs; #From Wilcoxon's rank sum test comparing URIs vs. LRIs.

ICD10: the International Classification of Disease version 10 (ICD10) codes.

For peer review only

For peer review only - http://bmjopen.bmj.com/site/about/guidelines.xhtml

Table 2. Results of the full generalized additive mixed models (GAMM) for upper respiratory tract infections and lower respiratory tract infections

	URIs				LRIs			
	**RR**	**LL**	**UL**	**P value**	**RR**	**LL**	**UL**	**P value**
Intercept	0.340	0.133	0.869	0.0251	1.421	0.381	5.303	0.6013
Lag 1 week incidence	1.202	1.159	1.245	0.0000	1.173	1.138	1.208	0.0000
Lag 2 week incidence	1.063	1.019	1.109	0.0048	1.053	1.015	1.092	0.0060
Lag 3 week incidence	0.999	0.957	1.042	0.9470	0.987	0.951	1.024	0.4861
Lag 4 week incidence	0.974	0.934	1.017	0.2328	0.998	0.962	1.035	0.9180
Temperature (°C)	1.017	0.996	1.040	0.1178	1.006	0.985	1.028	0.5748
Lag 1 week temperature	0.990	0.966	1.015	0.4480	0.976	0.953	0.998	0.0348
Lag 2 week temperature	1.019	0.994	1.045	0.1389	1.007	0.984	1.030	0.5620
Lag 3 week temperature	1.010	0.985	1.035	0.4470	1.019	0.995	1.043	0.1186
Lag 4week temperature	1.012	0.990	1.035	0.2709	1.015	0.993	1.038	0.1722
Humidity (%)	0.998	0.993	1.004	0.5619	0.998	0.993	1.004	0.5506
Lag 1 week humidity	1.009	1.003	1.014	0.0051	0.997	0.991	1.002	0.2716
Lag 2 week humidity	1.002	0.996	1.007	0.6000	1.001	0.995	1.006	0.7603
Lag 3 week humidity	0.990	0.984	0.996	0.0008	1.000	0.995	1.006	0.9237
Lag 4 week humidity	1.005	0.999	1.010	0.1024	1.000	0.994	1.005	0.8610
Rainfall (mm)	1.000	0.997	1.003	0.9670	1.001	0.998	1.004	0.5615
Lag 1 week rainfall	1.001	0.997	1.004	0.7145	1.004	1.001	1.007	0.0073
Lag 2 week rainfall	1.002	0.998	1.005	0.2979	1.000	0.998	1.003	0.7548
Lag 3 week rainfall	1.006	1.003	1.009	0.0005	1.001	0.998	1.004	0.4927
Lag 4 week rainfall	0.997	0.994	1.001	0.1163	0.999	0.997	1.002	0.6949
Water level (cm)	0.999	0.997	1.000	0.1519	0.999	0.997	1.000	0.0751
Lag 1 week water level	1.001	0.999	1.002	0.2120	0.998	0.997	1.000	0.0111
Lag 2 week water level	0.999	0.998	1.001	0.2488	1.000	0.998	1.001	0.7821
Lag 3 week water level	1.000	0.998	1.001	0.7223	1.000	0.999	1.002	0.9576
Lag 4 week water level	1.003	1.002	1.005	0.0000	1.001	1.000	1.003	0.1473
Time trend (week)	1.092	1.022	1.166	0.0096	1.080	1.006	1.159	0.0345

Results of fixed effects (with only the results of lags up to 4 weeks) are shown.

P values for seasonal cycles from the full GAMM of URIs=0.5177 and LRIs<0.0001 respectively.

Adjusted R-squared of the full GAMM for URIs=0.72 and LRIs=0.88 respectively.

RR: rate ratio; LL and UL: lower and upper limit of 95% confidence interval.

For peer review only

For peer review only - http://bmjopen.bmj.com/site/about/guidelines.xhtml

Figure legends

Figure 1. Maps for empirical Bayesian estimated incidence rates of acute respiratory tract infections and other diseases at ward level

a) Ward level empirical Bayesian estimated incidence rates (EBR) per 1,000 population for ARIs with locations of the 3 study hospitals in green, b) ward level EBR for other diseases rather than ARIs. CH1: Children Hospital 1, CH2: Children Hospital 2; HTD: Hospital for Tropical Diseases. District names and district boundaries (grey) are shown. Rural districts are marked with *.

Figure 2. Plots of the citywide weekly incidence time series for upper respiratory tract infections and lower respiratory tract infections from the generalized additive mixed model (GAMM)

Upper panels (a): smoothed seasonal cycles (red line), time trend (blue line) from the basic Poisson GAMM model, fitted values (green line) from the full Poisson GAMM model and observed values (black line) of citywide weekly incidence (number of cases per 1000 population) of URIs. Lower panels (b): smoothed seasonal cycles (red line), time trend (blue line) from the basic Poisson GAMM model and fitted values (green line) from the full Poisson GAMM model and observed values (black line) of citywide weekly incidence of LRIs.

Figure 3. Time series of city wide weekly incidence of upper respiratory tract infections and lower respiratory tract infections versus weekly climatic covariates

Left panel: time series of weekly URI and LRI incidence and weekly average climatic covariates. Right panel: Scatter plots with fitted lines and 95% confidence interval from univariate Poisson generalized linear model for weekly URI or LRI incidence vs. each climatic covariate.

For peer review only - http://bmjopen.bmj.com/site/about/guidelines.xhtml

Supplementary Figures

Figure S1. Maps for empirical Bayesian estimated incidence rates of acute respiratory tract infections by year at ward level

District labels are shown. 1-12: district 1-12; 13-24: district Binh Chanh, Binh Tan, Binh Thanh, Can Gio, Cu Chi, Go Vap, Hoc Mon, Nha Be, Phu Nhuan, Tan Binh, Tan Phu, Thu Duc respectively. Locations of study hospitals are shown in green. *Rural districts.

Figure S2. Maps for empirical Bayesian estimated incidence rates of upper respiratory tract infections and lower respiratory tract infections at ward level

a) Empirical Bayesian estimated incidence rates (EBR) per 1,000 population of URIs with locations of hospitals in green, b) EBR of LRIs per 1000 population with locations of hospitals in green. CH1: Children Hospital 1, CH2: Children Hospital 2; HTD: Hospital for Tropical Diseases. District names and district boundaries (grey) are shown. *Rural districts.

Figure S3. Diagnostic plots of the full-generalized additive mixed models (GAMM) for upper respiratory tract infections and lower respiratory tract infections

Left panel: URIs; right panel: LRIs.

Figure S4. Autocorrelation function and partial autocorrelation function of the residuals of the full generalized additive mixed models (GAMM) for upper respiratory tract infections and lower respiratory tract infections (LRIs)

Left panel: URIs; right panel: LRIs.

For peer review only

For peer review only - http://bmjopen.bmj.com/site/about/guidelines.xhtml

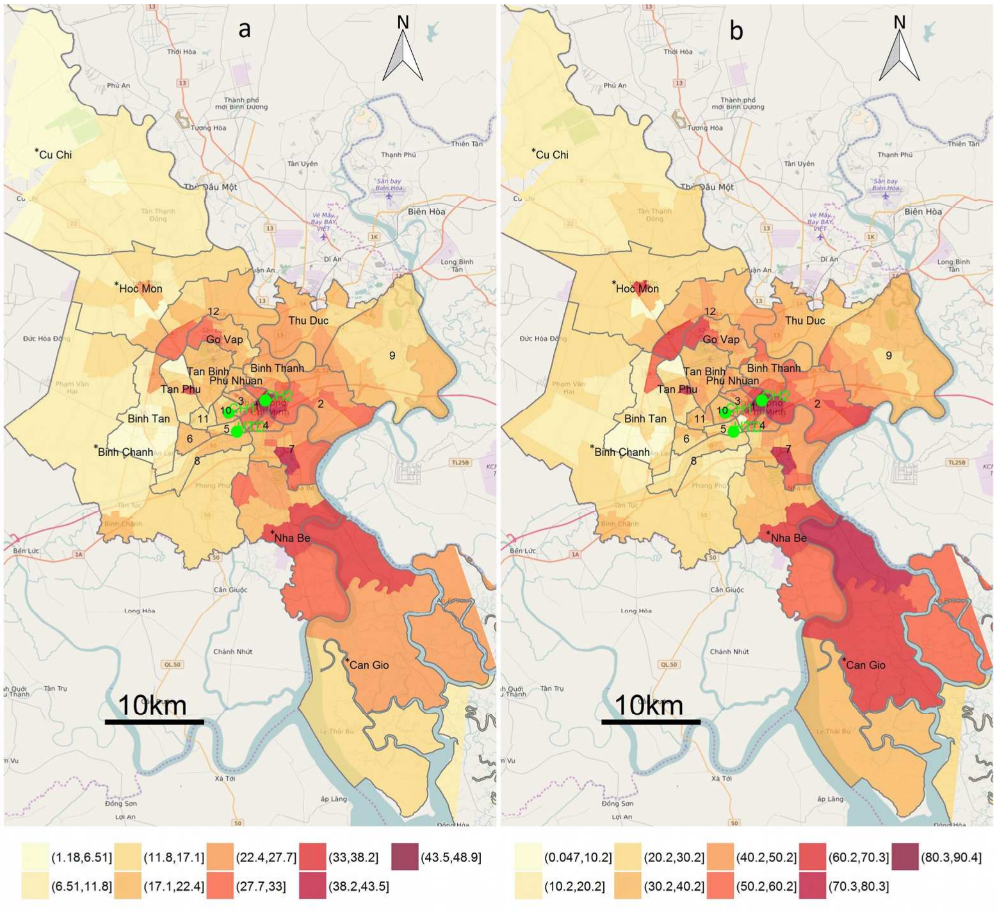

Figure 1

180x164mm (300 x 300 DPI)

only

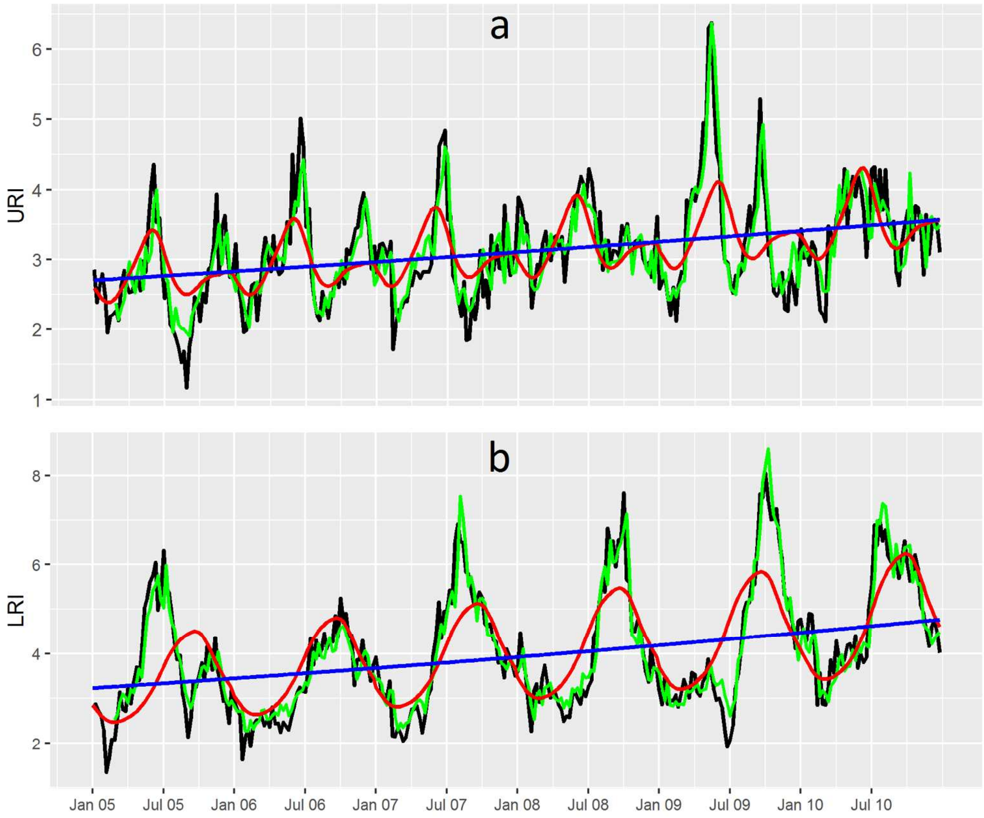

Figure 2

177x152mm (300 x 300 DPI)

For peer review only

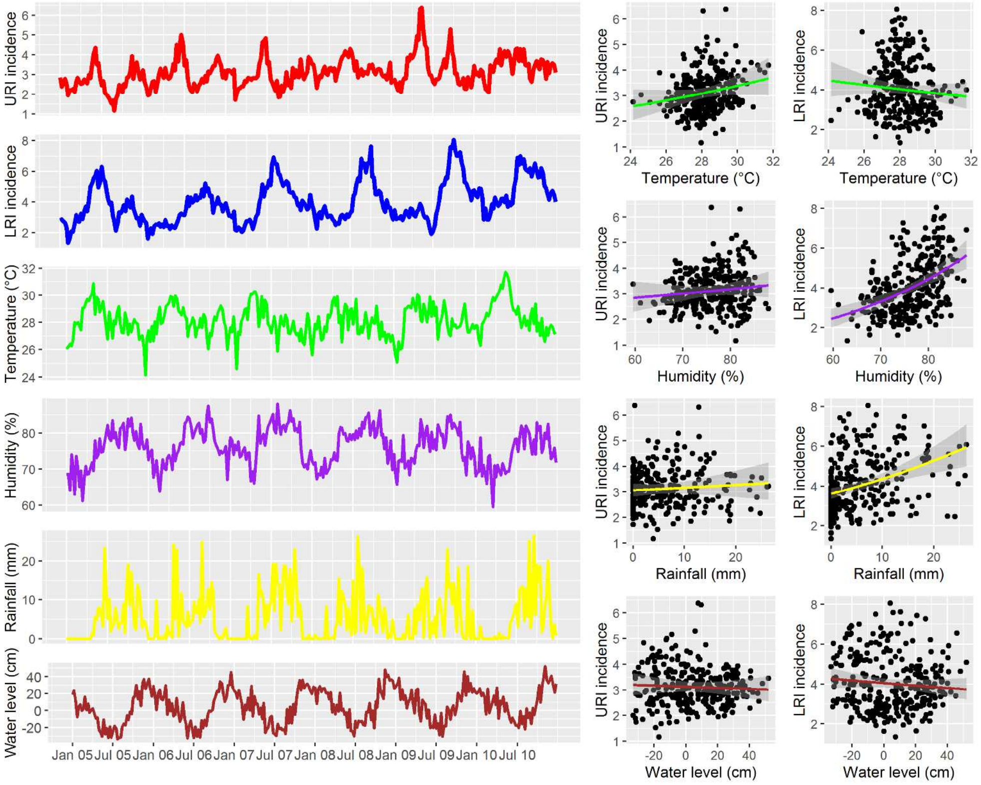

Figure 3

256x203mm (300 x 300 DPI)

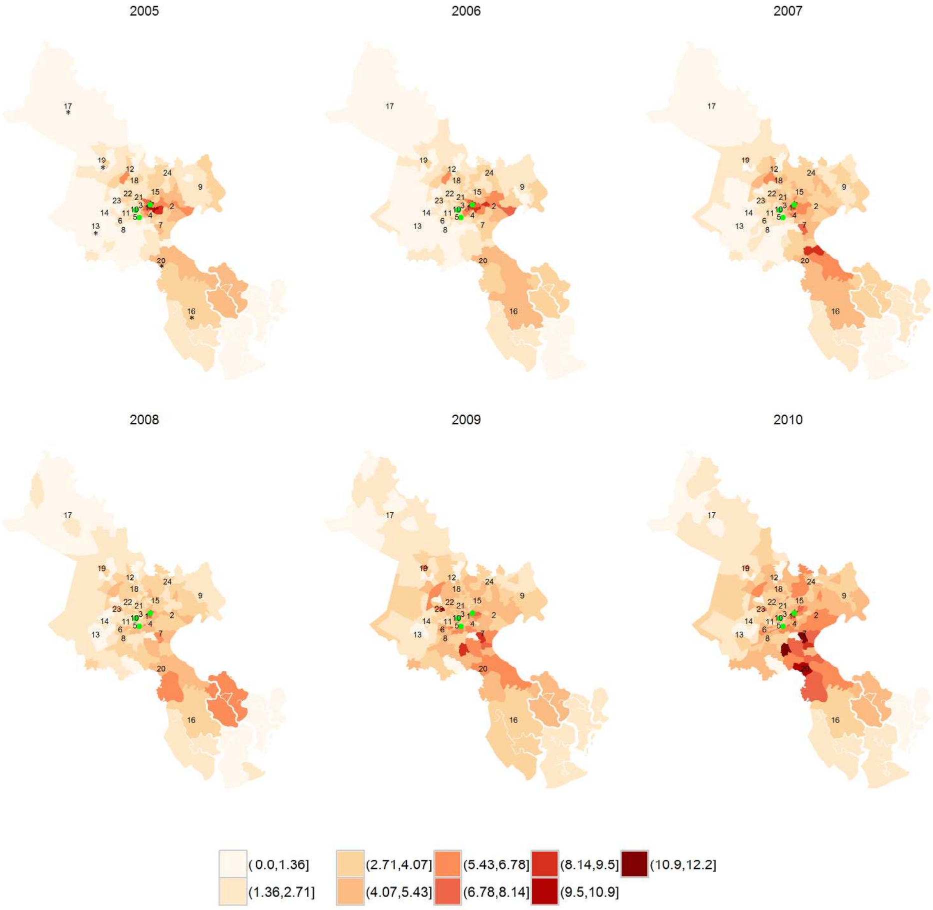

Figure S1

254x254mm (300 x 300 DPI)

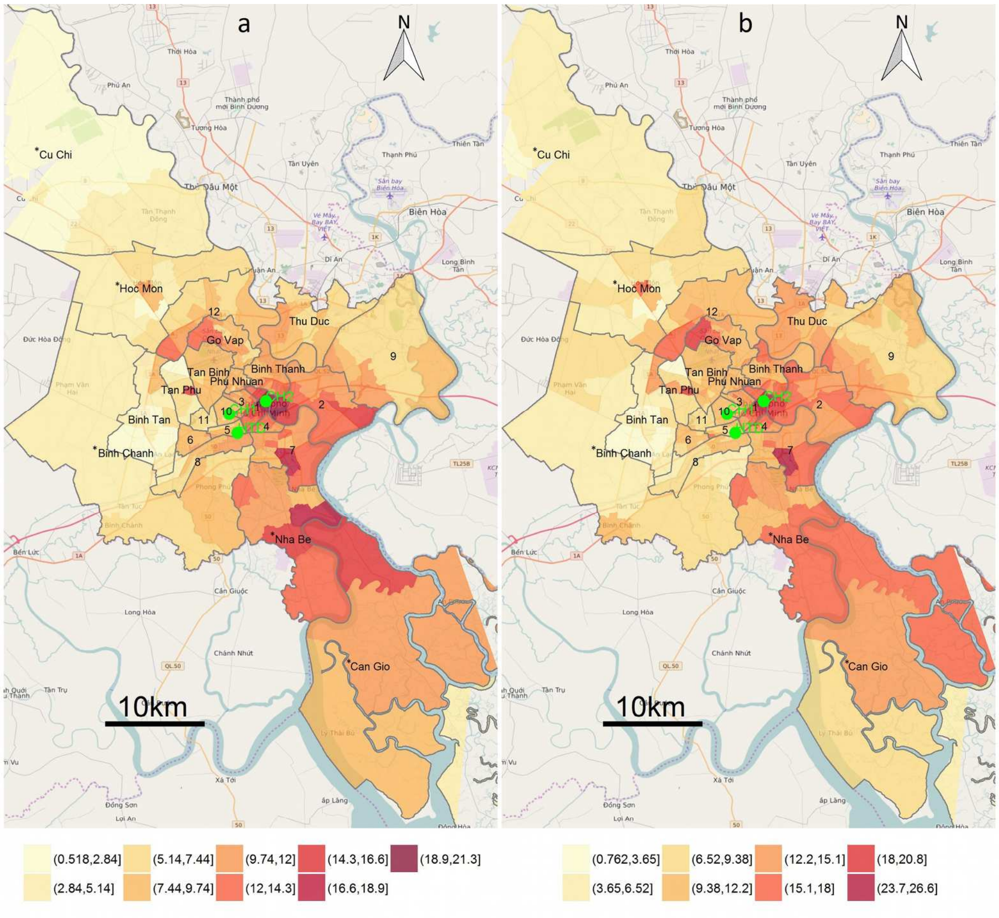

Figure S2

180x164mm (300 x 300 DPI)

For peer review only

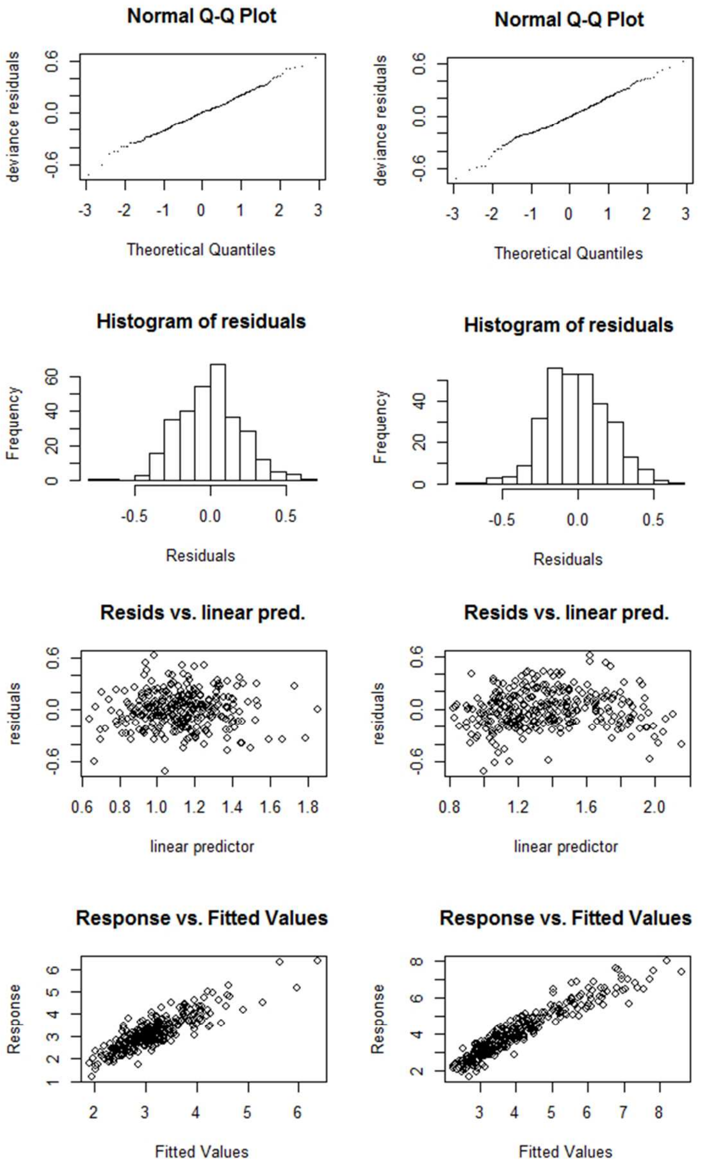

Figure S3

49x83mm (300 x 300 DPI)

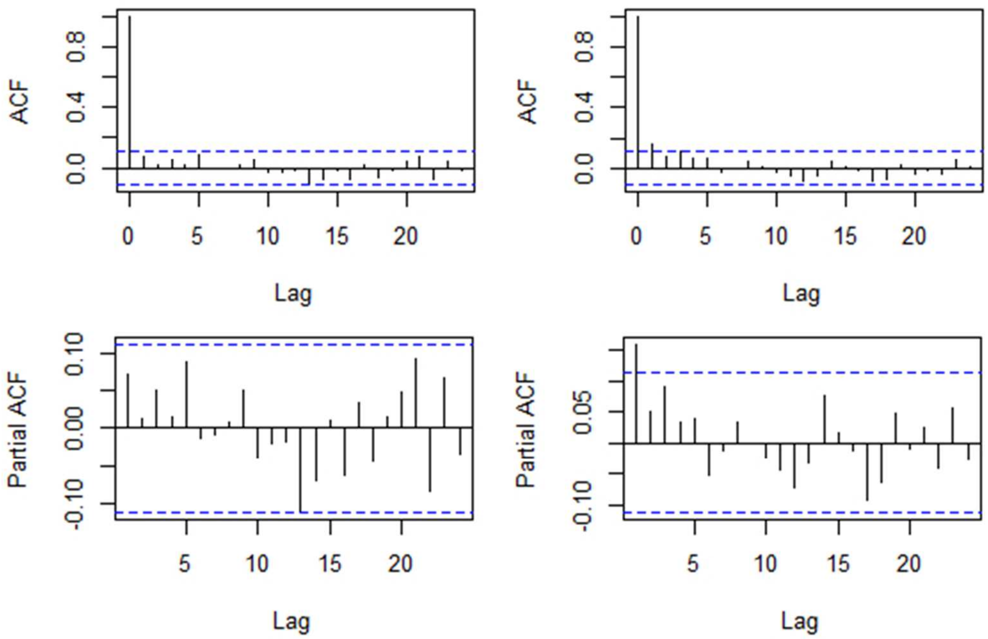

Figure S4

47x33mm (300 x 300 DPI)

For peer review only
